# In Vitro and Randomized Controlled Clinical Study of Natural Constituents’ Anti-HPV Potential for Treatment of Plantar Warts Supported with In Silico Studies and Network Analysis

**DOI:** 10.3390/ph17060759

**Published:** 2024-06-10

**Authors:** Nourhan Hisham Shady, Fatma Alzahraa Mokhtar, Hend Samy Abdullah, Salah A. Abdel-Aziz, Soad A. Mohamad, Mohamed S. Imam, Sherin Refat El Afify, Usama Ramadan Abdelmohsen

**Affiliations:** 1 Department of Pharmacognosy, Faculty of Pharmacy, Deraya University, Universities Zone, New Minia 61111, Egypt; norhan.shady@deraya.edu.eg; 2 Center for Research and Sustainability, Deraya University, Universities Zone, New Minia 61111, Egypt; 3 Fujairah Research Centre, Sakamkam Road, Sakamkam, Fujairah 0000, United Arab Emirates; drfatmaalzahraa1950@gmail.com; 4 Department of Pharmacognosy, Faculty of Pharmacy, El Saleheya El Gadida University, El Saleheya El Gadida, Sharkia 44813, Egypt; 5 Faculty of Pharmacy, Deraya University, Universities Zone, New Minia City 61111, Egypt; hend.samy_1180443@student.deraya.edu.eg; 6 Department of Pharmaceutical Chemistry, Faculty of Pharmacy, Deraya University, Universities Zone, New Minia 61111, Egypt; salah.abdelaziz@deraya.edu.eg; 7 Department of Pharmaceutical Medicinal Chemistry, Faculty of Pharmacy, Al-Azhar University, Assiut 71524, Egypt; 8 Department of Clinical Pharmacy, Faculty of Pharmacy, Deraya University, Universities Zone, New Minia 61111, Egypt; soad.ali@deraya.edu.eg; 9 Department of Clinical Pharmacy, College of Pharmacy, Shaqra University, Shaqra 11961, Saudi Arabia; 10 Department of Clinical Pharmacy, National Cancer Institute, Cairo University, Fom El Khalig Square, Kasr Al-Aini Street, Cairo 11796, Egypt; 11 Department of Pharmacology and Toxicology, Faculty of Pharmacy, Alsalam University, Kafr alzayat, Algharbia 31611, Egypt; sherinelafify@gmail.com; 12 Department of Pharmacognosy, Faculty of Pharmacy, Minia University, Minia 61519, Egypt

**Keywords:** *Moringa olifera*, *Nigella sativa*, *Musca accuminata*, HPV, network pharmacology, antiviral

## Abstract

The aim of this study is to evaluate the anti-HPV potential of a *Moringa olifera* Lam seed, *Nigella sativa* L. seed, and *Musa Acuminata* peel herbal mixture in the form of polymer film-forming systems. A clinical trial conducted in outpatient clinics showed that the most significant outcome was wart size and quantity. Compared to the placebo group, the intervention group’s size and number of warts were considerably better according to the results. Chemical profiling assisted by LC-HRMS led to the dereplication of 49 metabolites. Furthermore, network pharmacology was established for the mixture of three plants; each plant was studied separately to find out the annotated target genes, and then, we combined all annotated genes of all plants and filtered the genes to specify the genes related to human papilloma virus. In a backward step, the 24 configured genes related to HPV were used to specify only 30 compounds involved in HPV infection based on target genes. CA2 and EGFR were the top identified genes with 16 and 12 edges followed by PTGS2, CA9, and MMP9 genes with 11 edges each. A molecular docking study for the top active identified compounds of each species was conducted in the top target HPV genes, CA2 and EGFR, to investigate the mode of interaction between these compounds and the targets’ active sites.

## 1. Introduction

Viral infections play an essential part in human diseases, and recent epidemics due to globalization and the ease of travel have highlighted their prevention as a key concern in protecting public health [1]. Human papilloma virus (HPV) is a common group of viruses that cause skin and genital wart problems in most people [2]. Human papilloma viruses involve different groups and have various epithelial tropisms and life-cycle approaches [3]. HPV infections are categorized as low-risk and high-risk infections [3]. Low-risk HPVs can progress to papillomatosis and, in rare cases, to cancer. However, high-risk HPV is less common but can lead to different types of human cancers, especially cervical cancer, anogenital cancers, and head and neck cancer [3]. The effectiveness of vaccines has been extremely high among young women who were HPV-seronegative before vaccination. However, the nonavalent vaccine can supply additional protection against HPV. Moreover, the notable decrease in HPV among vaccinated women compared with unvaccinated women shows the vaccine to be highly effective [4]. Plantar warts are one type of HPV infection that is caused by an infection in the outer layer of skin on the soles of the feet or hands [5]. When the virus enters through small fractures, wounds, or other vulnerable areas on the bottom of the infected limb, warts form. Plantar warts, which are tiny, scaly growths that appear on the feet or hands, can persist for several years in adults and between a few months and two years in youngsters if untreated [6]. Warts frequently appear on the balls and heels of the feet, as these areas experience the most pressure. Even though these warts are benign, they can cause pain, difficulties walking, and high chances of transmission [7,8]. Patients with warts seek a durable therapy to avoid recurrent visits to physicians. Cryotherapy, electrocoagulation, laser surgery, topical keratolytic, topical antimetabolite drugs, and topical herbal extracts for wart treatment have all been developed. The effectiveness of each of these treatments varies greatly from patient to patient, but recurrence is frequent [9]. Effective therapies remain confronted with difficulties in patient compliance. Herbal medicines and purified natural ingredients are a rich resource for the creation of new antiviral drugs [1]. Because synthetic antiviral treatments are not available against most viral agents, all efforts have been directed toward the discovery of novel pharmaceuticals and complementary/alternative therapies derived from various herbal formulations. Medicinal plants have biochemical and bioactive components that can target specific viruses or heal or prevent a variety of viral illnesses and infections [10,11,12]. Natural herbal formulations and compounds derived from plants are widely used as a rich resource for the development of novel antiviral drugs against a variety of viruses, including coronaviruses, influenza viruses, human immunodeficiency viruses, herpes simplex viruses, severe acute respiratory syndrome (SARS) virus, Middle East respiratory syndrome (MERS) virus, and hepatitis B and C viruses [13]. Our study is focused on natural herbal formulas made of *Nigella sativa* (*N. sativa*), *Musa Acuminata* peels (*Musaceae*), and *Moringa oleifera* (*Moringaceae*) that have antiviral activity. *Nigella sativa* (*N. sativa*) is a member of the *Ranunculaceae* family. Seeds and oils have a long history of use in various systems of medicine and nutrition. The presence of thymoquinone, a significant bioactive ingredient of the essential oil, is responsible for the majority of this plant’s medicinal effects [14]. *N. sativa* contains a variety of phytoconstituents and derived compounds that have a wide range of biological effects, including antioxidant, anti-inflammatory, antibacterial, anti-fungal, anti-parasitic, anti-protozoal, antiviral, cytotoxic, anticancer, and neuro-, gastro-, cardio-, hepato-, and nephroprotective properties [15,16]. Moreover, traditional medicine uses banana plants to treat viral infections such as measles and chickenpox [17]. Earlier studies have shown that banana peels contain vitamins C, E, and B6 which act as antioxidants, especially vitamin C [18]. Furthermore, *Moringa oleifera* (*Moringaceae*) has been used in many traditional therapies and pharmacopoeias to treat a wide range of medical disorders such as malaria, diabetes, skin infections, tuberculosis, anemia, headaches, epilepsy, and sexually transmitted infections, as well as for wound healing [19]. The herb is widely used in African traditional medicine to treat AIDS and related secondary diseases caused by HIV. It shows substantial antiviral activity against viruses such as HIV, HSV, HBV, EBV, FMDV, and NDV [20]. Regarding formulations, several transdermal drug delivery methods and drug targeting formulations at the desired concentration have been studied in previous studies [21]. Precise and efficient film-forming methods have considerable potential for controlling medication distribution through the skin, attributable to the advantages of both films and hydrogels. Modifying plasticizers, additives, film-forming polymers, and model pharmaceuticals in formulations have all been investigated as ways to modify drug release through the skin [22,23,24]. This provoked us to investigate the anti-HPV activity of a mixture of *Moringa oleifera*, *Nigella sativa*, and *Musa acuminata* peels supported by *metabolomic* analysis of the three parts to reveal the phytochemical constituents of the three plants. Additionally, a network pharmacology study was performed to determine the genes annotated by the plant mixture involved in HPV infection. Finally, in the current study, we aimed to produce a novel anti-HPV herbal mixture in polymer film-forming systems (PFFs) as a promising treatment for plantar warts by using a procedure that includes human volunteers in clinical trials.

## 2. Results and Discussion

**Figure 1:**
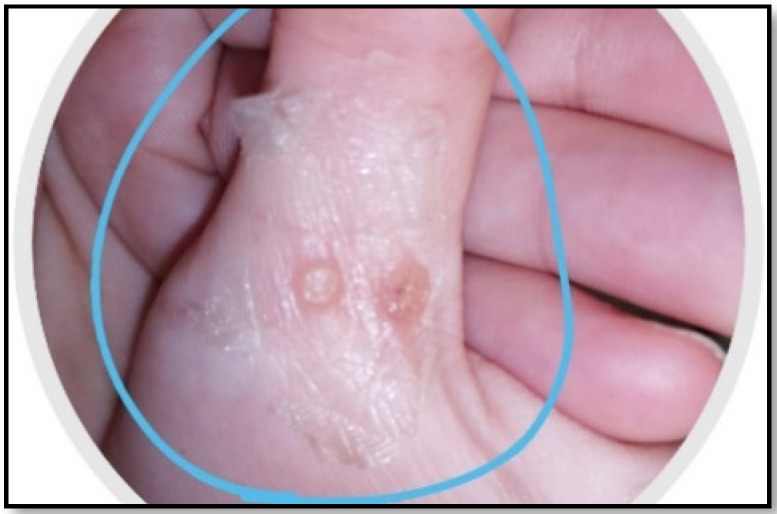
Organoleptic characteristics of PFF showing color, thickness, and sight.

A synergistic botanical blend was prepared from *Moringa oleifera*, *Nigella sativa*, and *Musa acuminata* peels methanolic extract with a ratio of 1:1:1. Then, the adhesive film, as an attractive method for a topical and transdermal drug delivery system, was prepared [24,25]. Chitosan and PVA together are known as a very compatible duo that produces good films in terms of its mechanical, morphological, and controlled release characteristics. The herbal PFF showed appropriate thickness for skin application (0.012 ± 0.05 mm) as it was clear and did not affect normal skin appearance, as shown in Figure 1. The folding endurance (70 ± 33) was sufficient for the application’s purpose and timing. The stickiness forces (0.8 ± 0.1 N) revealed a beneficial property that allowed the medication to stay where it was applied for the specified amount of time. Regarding the viscosity of the solution, it was 100 ± 2 cp, which signifies the flowability of the solution on application.

**Figure 2:**
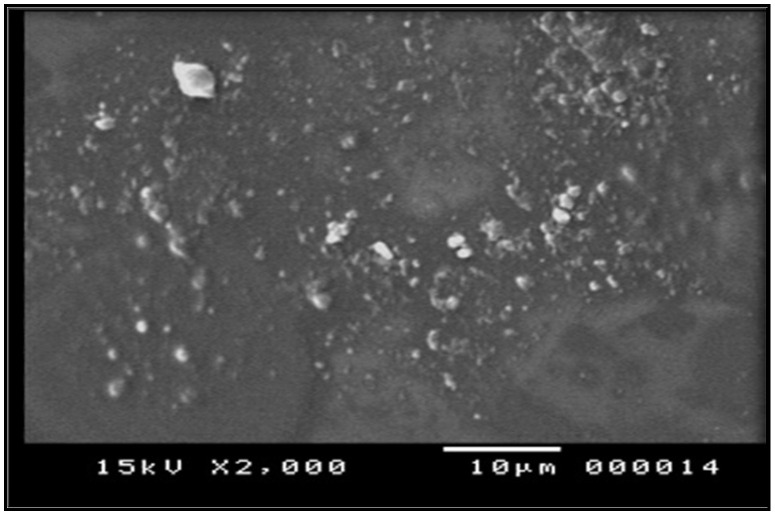
Electro-microscopic scanning of herbal film.

Morphological characterization of prepared film using SEM revealed a smooth background of the film components with small particles well distributed on this base, as seen in Figure 2. The figure shows good distribution of the extract that imparts good loading abilities of the polymer mixture [26].

**Figure 3:**
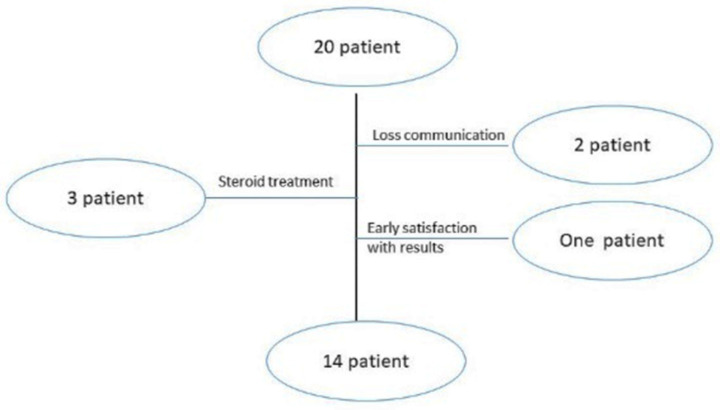
Patient flowchart of clinical assessment.

A relevant variation in patient demographics and clinical history was detected in plantar wart recurrence; about 30% of participants suffered from wart return that is reported as part of a special category already suspected for wart return [2]. The participants’ mean age was 45 ± 3 years, with a mean history of warts of 30 ± 7 months; females comprised the highest percentage of patients that may return due to their high emotional problems [27]. Figure 3’s participant flowchart illustrates the number of patients included in the clinical study. There were initially 20 patients included; two participants missed the next schedule due to a breakdown in communication, and three others took oral steroids while undergoing therapy, but steroids can interfere with erythema and pain scoring [27]. The last dropout was a participant who stopped enrolling after five weeks because he was delighted with the outcomes.

**Figure 4:**
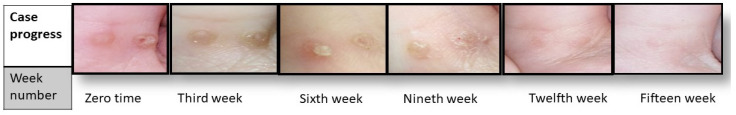
Case progress over 15 weeks.

The first clinical examination revealed that the participants had an average of 4 ± 0.4 warts with a diameter of 1.1 ± 0.2 mm. Progress in the clinical signs was supported by episodic photographing showing a reduction in warts remission (number and size), as seen in Figure 4. After fifteen weeks, the results significantly differed between the intervention and placebo group in terms of wart size (*p* < 0.01) and number (*p* < 0.05).

**Figure 5:**
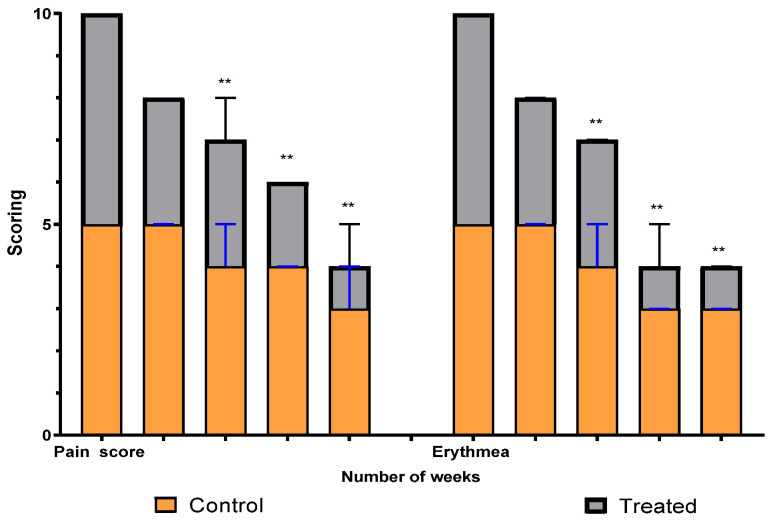
Erythema and pain scoring over fifteen weeks in study groups. ** means significant difference between treated group and control.

A comparison between the two groups over 15 weeks in terms of pain and erythema scores using the VAS method is illustrated in Figure 5. The graph shows a significant difference between the intervention (treated) group and the control group in scores that started at the third week and continued to weeks nine and fifteen. Erythema differs, with a high significant average score, between the two groups (*p* = 0.0019), while the pain average score shows a significant difference (*p* = 0.008).

### 2.1. LC-MS Assisted Dereplication of the Chemical Constituents in Nigella sativa Seed, Musca accuminata Peels and Moringa olifera Seed Extracts

Metabolomic profiling assisted by HR-LCMS analysis of the crude extract *Nigella sativa* seed (Table S1, Figures S7–S9) led to the identification of a wide range of secondary metabolites belonging to different phytochemical classes such as Nigellimine (**1**) [28], Nigellimine N-oxide (**2**) [29], 3,7-Dimethyl-6-octen-1-ol (**3**) [30], Nigeglanine (**4**) [31], Salfredin B_11_ (**5**) [32], Nigellicine (**6**) [33], 6-Octadecenoic acid (**7**) [34], Nigellidine (**8**) [35], Nigellidine 1-O-Sulfate (**9**) [36], Nigellamine C (**10**) [37], Nigellamine A_4_ (**11**) [37], Nigellamine A_3_ (**12**) [37], Nigellamine A_1_ (**13**) [38], Nigellamine A_5_ (**14**) [37], and Nigellamine B1 (**15**) [38]. Furthermore, profiling of the crude extract of *Musa Acuminata* peel resulted in the identification of several metabolites with different chemical classes such as 28-Hydroxy-7-octacosanone (**16**) [39], musabalbisiane A **(17)** [40], 2-hydroxyperinaphthenone (**18**) [41], naproxen (**19**) [42], 4-phenyl-1H,3H-naphtho [1,8-cd]pyran-1,3-dione (**20**) [41], 2-methoxy-9-phenyl-1H-phenalen-1-one (**21**) [41], musafluorone (**22**) [43], 9-(4-Hydroxyphenyl)-2-methoxy-1H-phenalen-1-one (**23**) [41], 9-(3,4-dimethoxyphenyl)-2-methoxy-1H-phenalen-1-one (**24**) [44], bisanigorufone (**25**) [45], anigorootin (**26**)**,** 4′-Hydroxyanigorootin (27) [46], 4′,4″-dihydroxyanigorootin (**28**) [46], Mc-FCC-56 (**29**) [47], and 24-methylenepollinastanone (**30**) [48]. Finally, *Moringa olifera* extract profiling resulted in the identification of several bioactive compounds: moringyne (**31**) [49], catechin (**32**) [50], quercetin (**33**) [50], kaempferol (**34**) [51], gallic acid (**35**) [52], *p*-Coumaric acid (**36**) [50], ferulic acid (**37**) [50], caffeic acid (**38**) [50], protocatechuic acid (**39**) [50], cinnamic acid (**40**) [50], ellagic acid (**41**) [51], vanillic acid (**42**) [50], moringine (**43**) [53], Kaempferol 3,7-diglycosides 3-O-[β-D-Glucopyranosyl-(1→2)-[α-L-rhamnopyranosyl-(1→6)]-β-D-glucopyranoside],7-O-α-L rhamnopyranoside (**44**) [54], 4-Hydroxybenzyl isothiocyanate O-α-L-rhamnopyranoside (**45**) [55], (4-Hydroxybenzyl) carbamic acid ester, O-α-L-rhamnopyranoside (**46**) [56], Rhamnose (**47**) [57], 4-Hydroxyphenylacetic acid amide, O-α-L-rhamnopyranoside (**48**) [57], 3,4-Dihydro-4,8-dihydroxy-3-methyl-1H-2-benzopyran-1-one, and (3*R*,4*S*)-form (**49**) [58].

### 2.2. Network Pharmacology and Gene Ontology Analysis

#### 2.2.1. Plant–Compounds Networks

The active compounds from each of the three plants under study were identified using LC/MS techniques, and these results were used to build simple networks that visualize each plant compound; the formed networks are *Musca accuminata*–compounds, *Moringa olifera*–compounds, and *Nigella sativa*–compounds, as illustrated as Figures S1, S2, and S3, respectively.

#### 2.2.2. Compounds–Targets Networks

The annotated genes identified by each compound from the three plants were obtained from the Swiss Target prediction and Binding DB databases; these genes were used to construct compound–gene networks, where the active identified compounds of each species were connected to corresponding annotated genes. Musca compounds–targets, Moringa compounds–targets, and Nigella compounds–targets networks were constructed (Figures S4, S5, and S6, respectively).

#### 2.2.3. Plant–Compounds–Genes Networks

**Figure 6:**
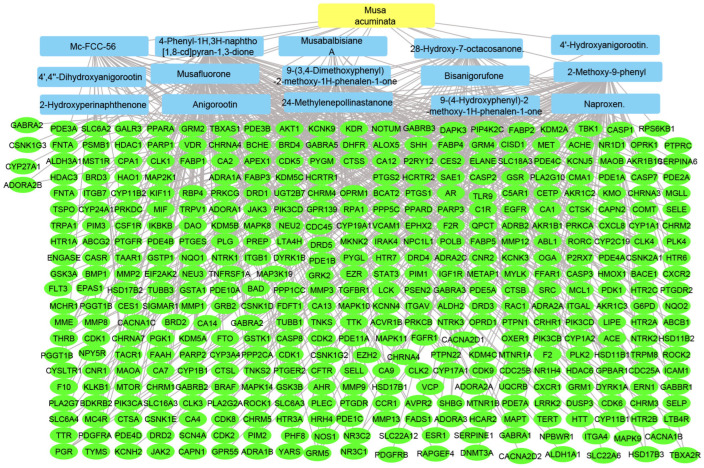
Musca–compounds–targets network: the merged network illustrates the identified compounds from *Musca accuminata* and the identified targets; the yellow rectangle represents the plant species (*Musca accuminata*), the blue rectangles represent the identified compounds, and the green ovals represent the identified targets.

**Figure 7:**
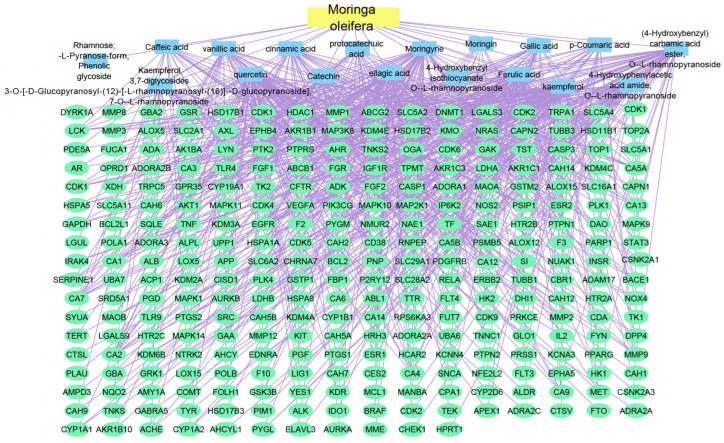
Moringa–compounds–targets network: the merged network illustrates the identified compounds from *Moringa olifera* and the identified targets; the yellow rectangle represents the plant species (*Moringa olifera*), the blue rectangles represent the identified compounds, and the green ovals shapes represent the identified targets.

**Figure 8:**
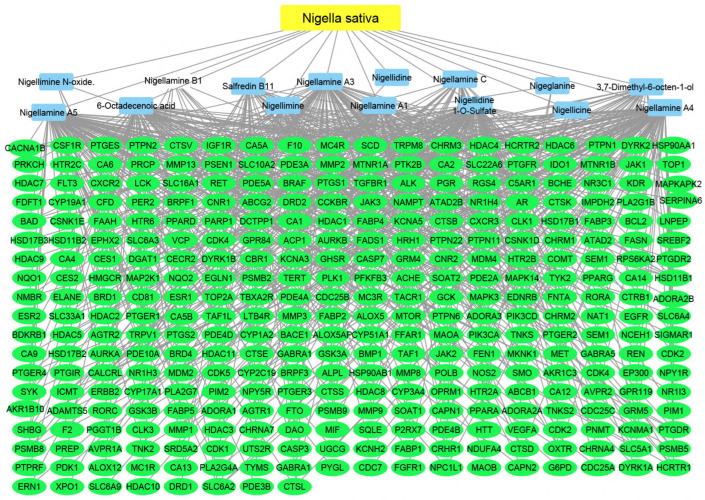
Nigella–compounds–targets network: the merged network illustrates the identified compounds from *Nigella sativa* and the identified targets; the yellow oval represents the plant species (*Nigella sativa*), the blue rectangles represent the identified compounds, and the green rectangles represent the identified targets.

Using the merging option in Cystoscope software, 3.9.0., for each species individually, a merged network that combines each plant with identified compounds and target hits was formed. The formed networks are Musca–compounds–targets (Figure 6), Moringa–compounds–targets (Figure 7), and Nigella–compounds–targets (Figure 8). Network analysis parameters are summarized in Table 1.

#### 2.2.4. Gene–HPV Infection Association

**Figure 9:**
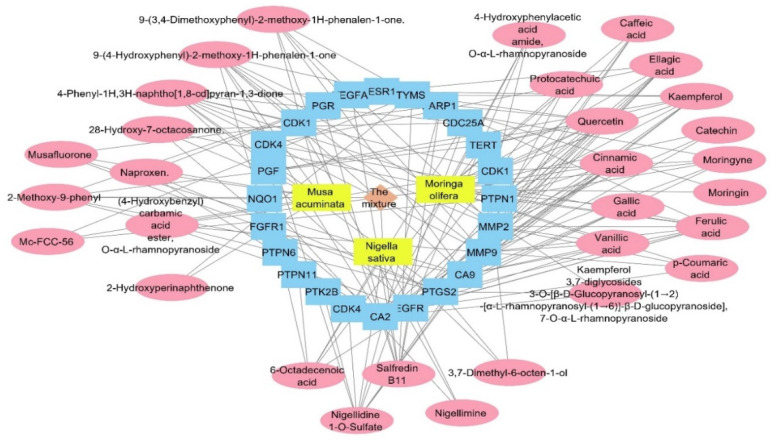
Total pharmacology network: the network combines the plant mixture formed of three plants in yellow rectangles (*Musca accuminata*, *Moringa olifera*, and *Nigella sativa*) with genes (in blue rectangles) related to the human papilloma virus and the compounds of these plants which identified the genes of the human papilloma virus (in pink oval shapes).

The target genes identified by the three plant species were combined and filtered to remove duplicates. The resulted genes were 686 targets. All of the genes were used as input data to the DisGeNET database to extract the gene–disease association between these genes and the human papilloma virus. All of the genes annotated by the three plants were combined and used as entry data in the DisGeNET database to figure out the gene–disease associations; the genes were associated with 48,273 diseases, while only 24 associations between human papilloma virus and 24 genes were obtained. Regarding the relation between the annotated genes and the human papilloma virus, we searched backwards to find its corresponding compounds, and a plant–compounds–genes–human papilloma virus network was constructed as a total pharmacology network (Figure 9). The formed network consisted of 58 nodes and 165. This network showed that the compounds involved in HPV are 30 compounds from the plant mixture distributed as follows: 5 compounds from *Nigella sativa*, 15 compounds from *Moringa olifera*, and 10 compounds from *Musca acuminate*. When we arranged the twenty-four genes related to HPV, CA2 and EGFR were the top identified genes, with 16 and 12 edges, followed by PTGS2, CA9, and MMP9 genes with 11 edges each. Carbonic anhydrase-II (CA2) is a cytosolic enzyme that is widely expressed in the endothelium of tumors’ neo-vessels. It plays a role in altering the PH microenvironment of tumors, and its targeted suppression is an extremely effective anti-angiogenic target therapy [57]. Moreover, the inhibition of CA2 is commonly associated with COX-2 inhibition, which suppresses the inflammatory cascade progression at the infective site and controls infection severity [58].

In terms of HPV induction of cancer, EGFR is one of the target genes that are overexpressed and triggered by the HPV protein (E1-E7), and is implemented in cancer cell proliferation, differentiation, and metastasis [59,60]. Targeting the inhibition of EGFR is essential to prevent cell apoptosis and/or proliferation through signaling the inhibition of MAPK/PI3K/STAT3 pathways [61,62].

### 2.3. Gene Enrichment Analysis

**Figure 10:**
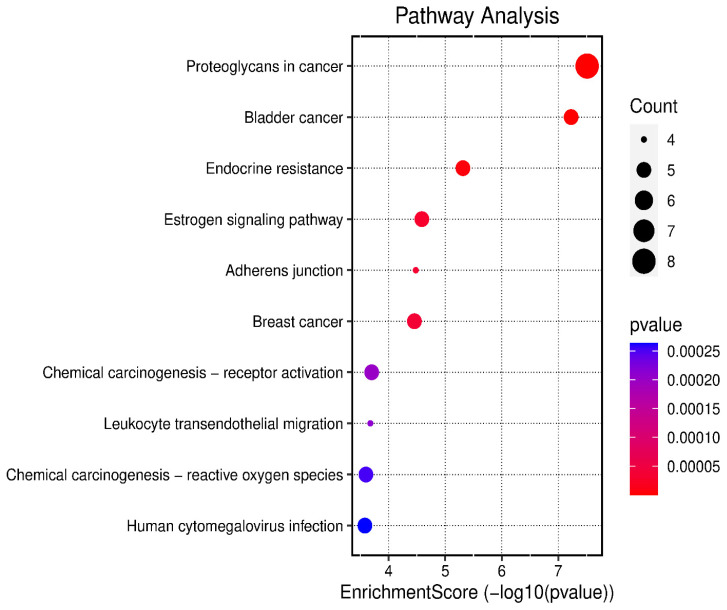
The top 10 biological KEGG pathways according to the enrichment score.

**Figure 11:**
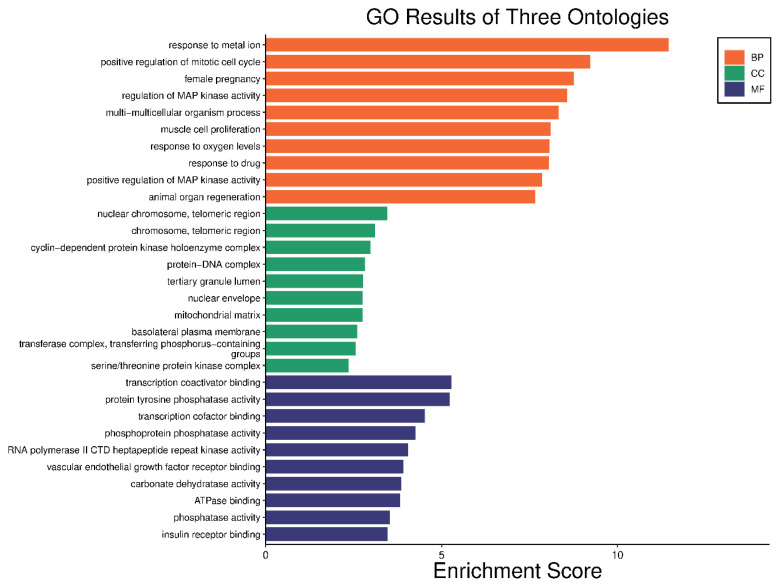
Gene enrichment analysis of gene set related to human papilloma virus; top 10 GO terms of biological process, cellular component, and molecular function.

The 24 genes that related to human papilloma virus were the only genes that were used as input data to perform gene enrichment analysis and pathway enrichment. A bioinformatics database illustrated the biological KEGG pathways according to the enrichment score. There were 58 identified biological pathways. The top pathways were proteoglycans in cancer (hsa05205), bladder cancer (hsa05219), and endocrine cancer (hsa01522) (Figure 10 and Table S2). The gene enrichment analysis of annotated genes found a total number of 1837 biological processes for these 24 genes; the top three biological processes were response to metal ions, positive regulation of mitotic cell cycles, and female pregnancy (Figure 11 and Table S3). The gene otology analysis in terms of cellular components identified 113 terms. The top three terms were nuclear chromosome and telomeric region (GO:0000784), chromosome and telomeric region (GO:0000781), and cyclin-dependent protein kinase holoenzyme complex (GO:0000307) (Figure 11 and Table S4). The identified molecular function revealed 167 terms. The top three were transcription coactivator binding (GO:0001223), protein tyrosine phosphatase activity (GO:0004725), and transcription cofactor binding (GO:0001221) (Figure 11 and Table S5).

A network pharmacology study was performed to determine the genes annotated by the plant mixture that are involved in HPV infection. There were only 24 genes involved in HPV infection among the 686 genes. The compounds involved in HPV are 30 compounds from the plant mixture distributed as follows: 5 compounds from *Nigella sativa*, 15 compounds from *Moringa olifera*, and 10 compounds from *Musca acuminate*. When arranging the twenty-four genes related to HPV infection, CA2 and EGFR were the top identified genes.

#### Molecular Docking

**Figure 12:**
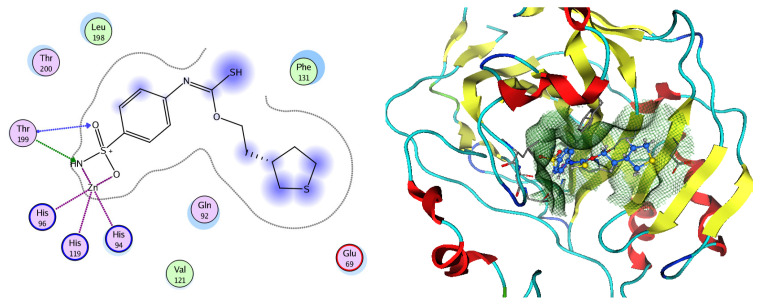
Two-dimensional and three-dimensional representation of the sulfonamide ligand (SUA) inhibitor interacting with CA-2 amino acid binding site.

**Figure 13:**
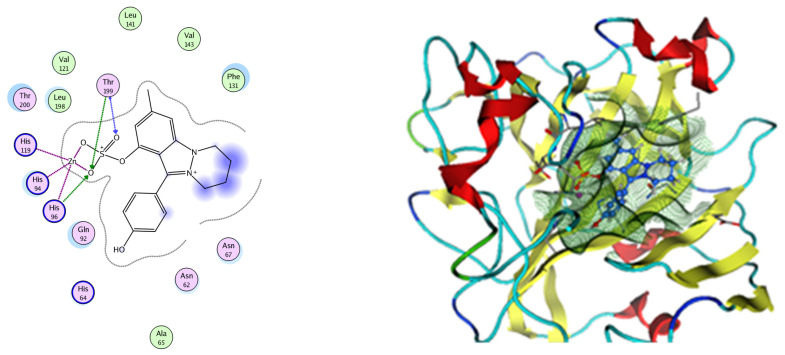
Two-dimensional and three-dimensional representation of Nigellidine 1-O-Sulfate interacting with CA-2 amino acid binding site.

Based on network pharmacology, the top target genes related to HPV from the combined three plant species compounds were identified. The top identified HPV genes were CA2 and EGFR. In the same manner, seven compounds, Nigellidine 1-O-Sulfate, Salfredin B11, ferulic acid, kaempferol, quercetin, 4-Phenyl-1H,3H-naphtho [1,8-cd] pyran-1,3-dione, and 9-(4-Hydroxyphenyl)-2-methoxy-1H-phenalen-1-one, were the sites where the top active identified compounds of each species connected to the corresponding annotated genes. These compounds were used for further molecular docking in the top target HPV genes, CA2 and EGFR, to investigate the binding interaction between the drugs and the targets active sites. We performed docking of the selected compounds into the crystal structures of the CA-2 and EGFR enzyme catalytic domains in a complex of CA-2 with sulfonamide inhibitor (SUA) [PDB ID code 3K34] [59] and EGFR with erlotinib [PDB ID code 1M17] [60], using Molecular Operating Environment MOE program version 2008.10 [61]. The most stable docking model was selected according to the best conformation score predicted by the MOE scoring function. The docking results are presented in Table 2 and Table 3 and Figure 12 and Figure 13. The docking protocol was validated through self-redocking of the co-crystalized ligands into the CA-2 and EGFR active sites, and the docking poses were compared with the initial enzymes’ pose using the root mean square deviation (RMSD). A sulfonamide inhibitor was docked into the CA-2 active site almost at the same position (RMSD = 1.001 Å), with a docking score of −10.17 kcal/mol^−1^, and showed the same orientations as previously reported. Also, erlotinib was docked almost at the same position in the EGFR active site (RMSD = 0.8392 Å), with a docking score of −11.02 kcal/mol^−1^, and showed the same orientations as previously. Carbonic anhydrases (CAs) are ubiquitous metallo-enzyme complexes with Zn^2+^. Human carbonic anhydrase 2 (hCA-2) is located at a large cone-shaped cavity containing a catalytic Zn^2+^ unit bonded with His94, His96, and His119 residues and a sulfonamide inhibitor is bound at the fourth position in a tetrahedral geometry [59]. hCA-2 has a wide distribution and is found in many different organs and cell types, and is thus involved in crucial physiological and pathological processes, such as respiration, the transportation of CO_2_/HCO_2_^−^ between metabolic tissues and the lungs, electrolyte secretion, pH and CO_2_ homeostasis, glaucoma, biosynthetic reactions, calcification, tumorigenicity, etc. [62,63].

**Figure 14:**
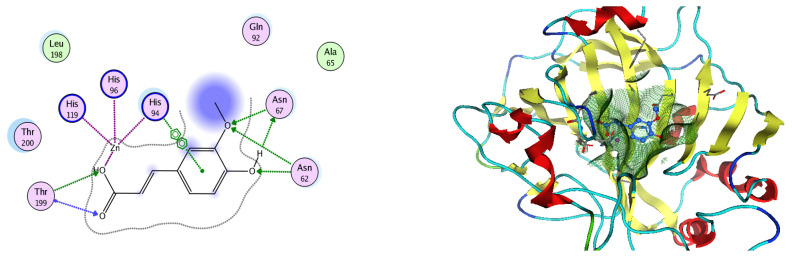
Two-dimensional and three-dimensional representation of ferulic acid interacting with CA-2 amino acid binding site.

**Figure 15:**
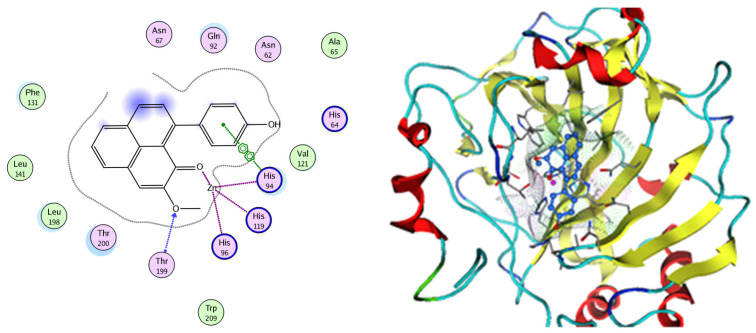
Two-dimensional and three-dimensional representation of 9-(4-Hydroxyphenyl)-2-methoxy-1H-phenalen-1-one interacting with CA-2 amino acid binding site.

Analysis of the docking data for the tested compounds showed that all of the seven docked compounds represent the most active identified compounds for the aforementioned genes that were docked in a parallel manner to the hCA-2 co-crystalized ligand, with docking scores ranging from −10.08 to −19.82 kcal/mol^−1^, and in a comparable manner to the co-crystalized sulfonamide ligand inhibitor. The catalytic Zn^2+^ plays a crucial role at the hCA-2 binding site as well as in the co-crystalized sulfonamide ligand inhibitor. Zn^2+^ interacts with all three hCA-2 histidine residues (His94, His96, and His119) through ionic bonds and with an additional one or two ionic bonds with ligand functionally (Figure 7, Figure 8, Figure 9 and Figure 10, Table 2). In the case of the sulfonamide ligand inhibitor, sulfonamide oxygen and nitrogen atoms form ionic bonds with Zn^2+^ and hydrogen bonds with THR199 (Figure 12, Table 2). Nigellidine 1-O-Sulfate is one of the most active identified compounds by the network and has a high docking score, representing *Nigella sativa*, which was docked in the same manner as the sulfonamide ligand inhibitor, with a high docking score of −19.82 kcal/mol^−1^, in which its sulfate oxygen atoms interact with Zn^2+^ through two ionic bonds and with THR199 and HIS96 through hydrogen bonds (Figure 13, Table 2). Ferulic acid is one of the most active identified compounds by the network and has a high docking score, representing *Moringa olifera*, which was docked in the same manner as the sulfonamide ligand inhibitor, with a docking score of −15.59 kcal/mol^−1^, in which its carboxylate oxygen interacts with Zn^2+^ through ionic bonding. Additionally, its carboxylate functionals interact with THR199 through hydrogen bonds, its phenolic hydroxyl and methoxy functionals interact with ASN62, ASN67, THR199, and HIS96 through hydrogen bonds, and its benzene ring interacts with HIS94 through Arene–Arene interactions (Figure 14, Table 2). 9-(4-Hydroxyphenyl)-2-methoxy-1H-phenalen-1-one is one of the most active identified compounds by the network and has a high docking score, representing musca acuminate, which was docked in the same manner as the sulfonamide ligand inhibitor, with a docking score of −14.28 kcal/mol^−1^, in which its carbonyl oxygen interacts with Zn^2+^ through ionic bonding. Additionally, its methoxy oxygen atom interacts with THR199 through hydrogen bonds, and its phenolic benzene ring interacts with HIS94 through Arene–Arene interactions (Figure 15, Table 2).

**Figure 16:**
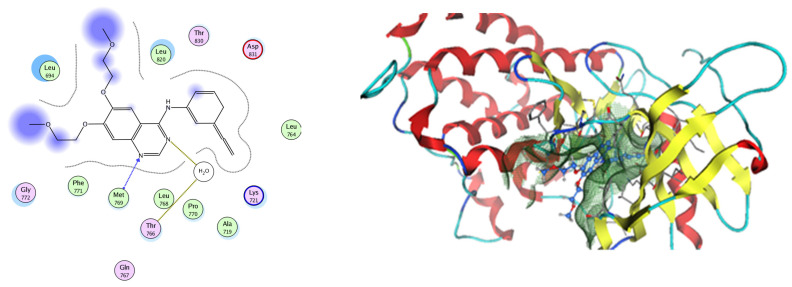
Two-dimensional and three-dimensional representation of erlotinib interacting with EGFR amino acid binding site.

**Figure 17:**
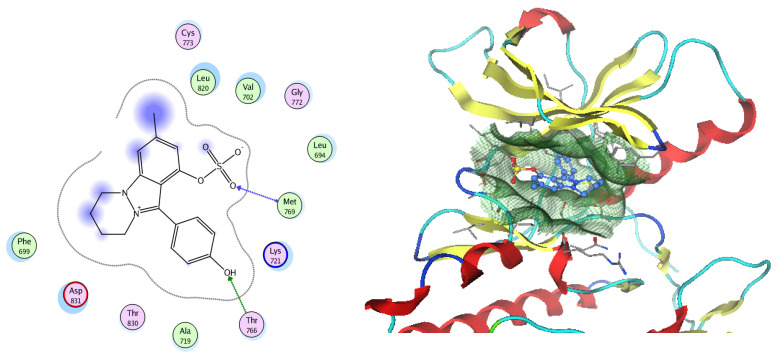
Two-dimensional and three-dimensional representation of Nigellidine 1-O-Sulfate interacting with EGFR amino acid binding site.

**Figure 18:**
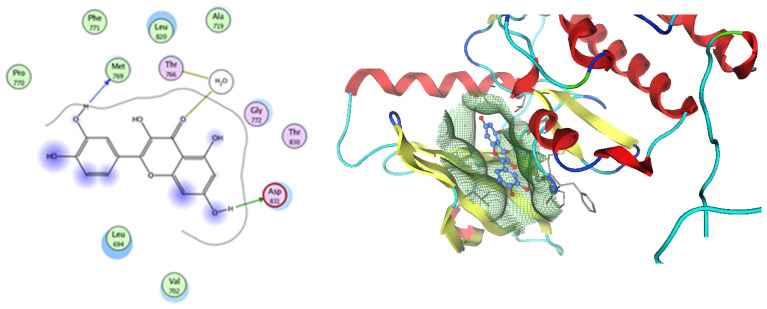
Two-dimensional and three-dimensional representation of quercetin interacting with EGFR amino acid binding site.

**Figure 19:**
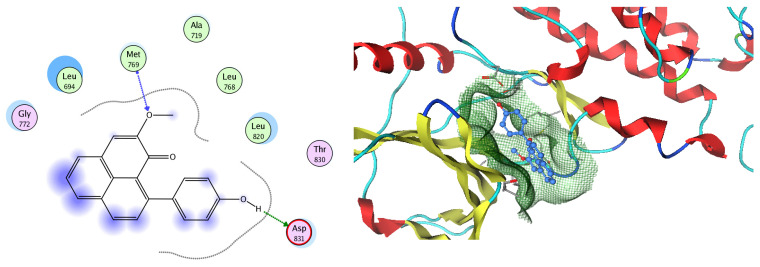
Two-dimensional and three-dimensional representation of 9-(4-Hydroxyphenyl)-2-methoxy-1H-phenalen-1-one interacting with EGFR amino acid binding site.

EGFR is a membrane tyrosine kinase receptor which is expressed in multiple organs and plays important roles in proliferation, survival, and differentiation in both development and normal physiology, as well as in pathophysiological conditions. Aberrant EGFR activation is a significant factor in the development and progression of multiple cancers, playing a key role in many inflammatory conditions, atherosclerosis, GIT secretion, and inflammatory bowel disease [64]. Analysis of the docking data for the tested compounds showed that all of the seven docked compounds were docked in a parallel manner to the EGFR co-crystalized ligand, with docking scores ranging from −8.93 to −14.78 kcal/mol^−1^, and in a comparable manner to the co-crystalized ligand inhibitor erlotinib, with a docking score of −11.02 kcal/mol^−1^ (Figure 16, Figure 17 and Figure 18, Table 3). Erlotinib interacts with active EGFR binding sites, showing H bonding interactions between quinazoline N1 and MET769, and H bonding interactions between quinazoline N3 and THr766 through a water bridge (Table 3, Figure 16). Nigellidine 1-O-Sulfate is one of the most active identified compounds by the network and has a high docking score, representing *Nigella sativa*, which was docked in the same manner as the ligand inhibitor erlotinib. It has docking score of −11.71 kcal/mol^−1^ in which its phenolic hydroxy interacts with THR766 through hydrogen bonds, and one of its sulfate oxygen atoms interacts with MET769 through hydrogen bonds (Table 3, Figure 17). Quercetin is one of the most active identified compounds by the network and has a high docking score, representing *Moringa olifera*, which was docked in the same manner as the ligand inhibitor erlotinib. It has a docking score of −14.78 kcal/mol^−1^ in which its chromen-4-one oxygen interacts with hydrogen bonds and THR766 through a water bridge, its 3-hydroxy-phenyl interacts with MET769 through hydrogen bonds, and its 5-hydroxy-chromen interacts with ASP831 through hydrogen bonds (Table 3, Figure 18). 9-(4-Hydroxyphenyl)-2-methoxy-1H-phenalen-1-one is one of the most active identified compounds by the network and has a high docking score, representing *Musca acuminate*, which was docked in the same manner as the ligand inhibitor erlotinib. It has a docking score of −11.10 kcal/mol^−1^ in which its methoxy oxygen interacts with MET769 through hydrogen bonds and its hydroxyphenyl interacts with ASP831 through hydrogen bonds (Table 3, Figure 19).

### 2.4. Pharmacokinetic Properties

The drug development process not only involves intensive searching to optimize the pharmacological properties of the lead compounds to ensure they are specific and more potent but also efficient delivery of these compounds to reach the sites of action. So, during the search by using the network for new compounds, we should consider their structural properties which are important for their pharmacokinetics in the human body. In particular, the molecular properties described in Lipinski’s rule of five and Veber’s rule determine if a compound has chemical and physical properties that would make it a likely orally active one. According to Lipinski’s rule of five, [65] oral bioavailability is likely to occur if at least three of the following rules are obeyed: M.w.t is not over 500 Da; there are no more than 5H bond donors; there are no more than 10H bond acceptors; and Clog P is not over 5. Additionally, according to Veber’s rule [62], compounds which match the two criteria of (1) being equal to or less than 10 rotatable bonds and (2) having a total polar surface area equal to or less than 140 Å^2^ (or equal to or fewer than 12 H bond donors and acceptors) are predicted to have good oral bioavailability. The data from the table for all of the seven tested compounds indicate that these compounds have good oral bioavailability, which obeys Lipinski’s rule of five and Veber’s rule for gastrointestinal absorption as shown in Table 4.

## 3. Material and Methods

### 3.1. Preparation of PFFs

Film-forming solutions were prepared by adding a chitosan 1% *w*/*v* solution in acetic acid to a polyvinyl alcohol 17% *w*/*v* solution and stirring the mixture overnight to ensure complete dissolution of the polymers. Having obtained a clear polymeric solution, maleic anhydride as a crosslinker and glycerol/propylene glycol as plasticizers were added separately with 2 h in between. After the addition of all additives, the herbal mixture was added and then the solution was stirred for another 24 h before use. The formulations were stored in glass vials sealed tightly with a siliconized rubber plug and an aluminum cap.

### 3.2. Evaluation of the PFFs

For assessment of the appropriateness of the film-forming solutions, the obtained solutions were evaluated for viscosity, mechanical properties, and stickiness of the outer surface [66]. A Brookfield DV-E Viscometer (Brookfield, MA, USA) equipped with an LV3 spindle was used to test the viscosities of the prepared solutions at a speed of 12 rpm. At 20 ± 0.5 °C for 5 min, the viscosity of each sample was measured, and the mean viscosity was calculated as the average of the three triplicates. The mechanical property assessment was performed by conducting a folding endurance experiment, as in previous studies, using the same force and recording the number of folds needed to break the film [67]. The stickiness of the prepared films was studied by using the modified Jolly balance method which determines the amount of force required to detach the films from a thin layer of rat skin [23]. A 20 g force was applied to the film for 30 s to create adhesion after moistening the side of the film surface that was facing the rat skin. The weight in grams required to remove the film from the surface of the skin provided a measurement of the adhesive strength, from which the adhesion force was calculated as follows: the adhesiveness of the samples was evaluated as the peak in the normal force (tension) in [N], related to the area, and expressed in [N∙cm^−2^]. The data are presented as means ± standard errors of the mean of the three samples.

### 3.3. Polymers’ Compatibility and Safety

PVA and chitosan co-polymers were selected as anti-HPV herbal extract delivery vehicles based on previous investigations. The co-polymers are well known for being biocompatible and non-toxic blends that are created through initiation and crosslinking with high shear [68]. Furthermore, the in vivo-confirmed wound healing characteristics of this special mixture make it favorable [69]. Furthermore, chitosan’s antifungal and antibacterial properties have been documented in numerous earlier studies, which contribute to the formulation’s prolonged shelf life [70]. It has been observed that using this mix of hydrogel dressings will speed up the healing process for burn injuries [71]. Thus, our study suggests that this combination is the safest and most efficient way to treat plantar warts in a new, more potent formulation form.

#### Scanning Electron Microscopy (SEM)

The morphology of the microstructure of the formulated films was studied using a scanning electron microscope (SEM) (JSM-IT 200, Leica, Germany) at accelerating voltages of 10 kV, 15 kV, and 20 kV. The samples were coated with an 8 nm thick layer of gold before the measurement by using an EM ACE200 sputter coater (Leica, Germany). The films’ top surfaces and fracture surfaces were analyzed.

### 3.4. Patients and Method

A prospective, single-center, interventional, parallel randomized study was conducted over a period of 8 months from August 2022 to January 2023 in outpatient clinics. Male and female patients who were diagnosed with plantar warts and aged more than 18 years were invited to comply with the protocol to be included in the study. Before initiating the study, ethical approval was obtained from the institutional ethical committee at Deraya University under number 1/2022 and recorded in clinicaltrials.gov under the number ID NCT05592041 [72]. The study excluded women who were pregnant, nursing, had other forms of warts, and had co-morbid illnesses such diabetes or immunosuppression disorders. All patients were enrolled once written informed consent was received. Participants underwent thorough systemic, dermatological, general physical, and clinical history examination, during which the main investigator documented the size (as determined by a vernier caliper), inflammation score, pain score, and number of warts found on each participant. After preliminary clinical evaluation, the study included 20 cases which were allocated into 2 groups (intervention and placebo) at random. The intervention group received herbal PFFs, whereas the placebo group received plain PFFs. In both groups, the treatment was prescribed twice a day for three months, with frequent clinical and photographic evaluations every three weeks [72].

The number of warts and the size were evaluated clinically by using the VAS method and dermato-photography; inflammation scoring was evaluated from 1 (the least) to 5 (the highest) by the main investigator and the average of three visits was calculated. Pain scoring was evaluated from 1 (the least) to 5 (the highest) based on the patients’ pain rating and the average of three visits was calculated.

The data were analyzed by using Statistical Package for GraphPad 3 using two-way RM ANOVA analysis. The quantitative data were presented as means, SDs, and ranges when their distribution was found to be parametric. The qualitative variables were presented as numbers and percentages. *p* > 0.05: nonsignificant (NS), *p* ≤ 0.05: significant (S), and *p* ≤ 0.01: highly significant (HS).

#### Plant Collections

The seeds of *Nigella sativa*, *Banana peels*, and the seeds of *Moringa oleifera* (*Moringaceae*) were collected in January 2023 from public nurseries in Minia Governorate, Egypt. Authentication of the plant was identified by Prof. Dr. Nasser Barakat, Department of Botany, Faculty of Science, Minia University, Minia, Egypt. A voucher specimen (Mn-ph-Cog-062) has been deposited in the Herbarium of Pharmacognosy Department, Faculty of Pharmacy, Deraya University, Minia, Egypt.

### 3.5. Metabolomic Analysis

LC-MS was carried out using a Synapt G2 HDMS quadrupole time-of-flight hybrid mass spectrometer (Waters, Milford, CT, USA). The sample (2 µL) was injected into the BEH C18 column, adjusted to 40 °C, and connected to a guard column. A gradient elution of mobile phase was used, starting from 100% water in 0.1% formic acid as solvent A to 100% acetonitrile in 0.1% formic acid as solvent B. MZmine 2.12 (San Diego, CA, USA) was employed for the differential investigation of MS data, followed by converting the raw data into positive and negative files in mzML format with ProteoWizard (Palo Alto, CA, USA).

### 3.6. Network Pharmacology and Gene Ontology Analysis

#### 3.6.1. Plant–Compounds Networks

The active compounds from each of the three plants under study were identified using LC/MS/MS techniques; the results of LC/MS/MS were used to construct the plant–compounds networks.

#### 3.6.2. Compounds–Targets Network

For the construction of these networks, chemical data were obtained for each compound from the PubChem database (https://pubchem.ncbi.nlm.nih.gov/) [73] (last accessed on 24 February 2023), and the Swiss Target Prediction database (http://www.swisstargetprediction.ch/result.php?job=215444691&organism=Homo_sapiens) [74] (last accessed on 28 February 2023) was used to find out the targets of each identified compound from *Musca accuminata*, *Moringa olifera*, and *Nigella sativa* related to the human species (*Homosapien*). We selected the top targets that were chosen in the Swiss Target Prediction database with a probability score > 0.

#### 3.6.3. Merged Networks

Merged networks were constructed for every plant species and all identified compounds. And we annotated the genes by combining the plant–compounds network and the corresponding cps–genes network.

#### 3.6.4. Genes–HPV Infection Association Network

The DisGenet (https://www.disgenet.org/) [75] (last accessed on 7 March 2023) online database was employed to figure out the target genes related to HPV infection.

#### 3.6.5. Complete Pharmacology Network

We combined the plants of the mixture with the genes involved in HPV infection and performed a backward search to find its related compounds and formed the complete pharmacology network. This network and previously formed networks were constructed, visualized, and analyzed using the software Cytoscape 3.9.0. (https://cytoscape.org/download.html, accessed on 25 August 2023) [76].

### 3.7. Gene Ontology and Enrichment Analysis

The gene ontology and enrichment analysis was performed on all genes of the identified compounds involved in HPV infection to find out the GO terms of the cellular components, molecular function, and biological processes that were affected by the annotated genes using a graphical gene set enrichment tool (http://www.bioinformatics.com.cn/basic_local_go_pathway_enrichment_analysis_122_en) [77] (accessed on 25 August 2023).

### 3.8. Molecular Docking

#### 3.8.1. CA-2

Molecular modeling calculations and docking studies were carried out using Molecular Operating Environment (MOE version 2008.10) [78]. For this purpose, the X-ray crystal structure of CA-2 in enzyme catalytic domains in a complex with the sulfonamide (SUA) inhibitor [PDB ID code 3K34] was obtained from the Protein Data Bank to prepare the protein for docking studies [59]. The enzyme was prepared for docking as follows: (1) we removed the excess water and ligand other than the sulfonamide inhibitor; (2) the enzyme was 3D-protonated, where hydrogen atoms were added at their standard geometry, surfaces and maps were computed, and the system was optimized; (3) docking of the co-crystallized ligand was performed to study the amino acid interactions, determine the binding energy score, and confirm the validity of the docking steps through determining the root mean square deviation (RMSD). Preparation of the seven chosen tested compounds for docking was achieved by building their 2D structures by using ChemDraw Ultra 8 and copying them to the MOE program and database formation. The test compounds were subjected to energy minimization with MOE until an RMSD gradient of 0.01 Kcal mol^−1^ A˚^−1^ was obtained with MMFF94X forcefield refinement. Further, we performed a conformational search for the energy-minimized tested compounds and chose the most energy-stable conformers for docking. Flexible ligand–rigid receptor docking of the most stable conformers was conducted with MOE-DOCK using triangle matcher as the placement method and London dG as a scoring function. The obtained poses were subjected to forcefield refinement using the same scoring function. Thirteen of the most stable docking models for each ligand were retained with the best conformation score.

#### 3.8.2. EGFR

Molecular modeling calculations and docking studies were carried out using Molecular Operating Environment (MOE version 2008.10) [78]. For this purpose, the X-ray crystal structure of EGFR tyrosine kinase in a complex with erlotinib (PDB ID code 1m17) was obtained from the Protein Data Bank to prepare the protein for docking studies [61]. The enzyme was prepared for docking as follows: (1) the enzyme was 3D-protonated, where hydrogen atoms were added at their standard geometry, surfaces and maps were computed, and the system was optimized; (2) docking of the co-crystallized ligand was performed as discussed previously with CA-2.

### 3.9. Pharmacokinetic Properties and Drug Likeness

Lipinski’s rule of five and Veber’s rule calculations were carried out using Molecular Operating Environment (MOE version 2008.10) [78]. The seven chosen tested compounds in their MOE database were assessed based on Lipinski’s rule of five through open computing, describing and selecting the codes needed by Lipinski’s rule of five, then using Veber’s rule, and pressing ok.

## 4. Conclusions

This study describes an improved approach for managing plantar warts attributed to causative HPV, which was verified based on photographs of the warts’ size and quantity, and pain and erythema scoring before and after treatment. It has been proven that a novel herbal blend in the form of polymer films can be an effective regimen for improving the skin quality of patients that are impaired by plantar warts. Furthermore, the chemical profiling of *Moringa olifera* seeds, *Nigella sativa* seeds, and *Musa acuminata* peels assisted by LC-HRMS (an HPLC system coupled to a high-resolution mass detector) led to the dereplication of 49 metabolites. Regarding the network pharmacology study, the mixture of the three species identified CA2 and EGFR as the top identified genes related to HPV infection in the studied gene set. Regarding the molecular docking studies, the most active identified compounds of each species showed good fitting and a comparable binding interaction with key amino acid residues bonded to native ligands for both CA2 and EGFR enzymes, the top target HPV genes. In this manner, Nigellidine 1-O-Sulfate, ferulic acid, kaempferol, quercetin, and 9-(4-Hydroxyphenyl)-2-methoxy-1H-phenalen-1-one showed binding energy scores in relation to the CA2 enzyme 1.5–2-fold greater compared to the native sulfonamide ligand (SUA) inhibitor. Quercetin showed a binding energy score in relation to the EGFR enzyme 1.4-fold greater compared to the native erlotinib ligand inhibitor. Supporting this NP study, the most active identified compounds of each species were further in silico studied to assess their gastrointestinal absorption by applying Lipinski’s rule of five and Veber’s rule analysis. The data indicated that this herbal mixture could be a future strategy to combat HPV infections.

## Figures and Tables

**Table 1:** A summary of the constructed networks for each plant species describing the source and target nodes of each network.

NO.	Network Name	Source Node/s	No. of Source Node/s	Target Node/s	No. of Target Node/s	Total No. of Nodes	No. of Edges	Characteristic Path Length	Network Centralization	Figure No.
1	*Musca accuminata*–compounds	*Musca accuminata*	1	Cpds.	15	16	15	1.875	1.0	Figure S1
2	*Musca accuminata*–compounds–genes	*Musca accuminata* compounds	15	Targets	412	427	645	3.507	0.231	Figure S4
3	*Musca accuminata*–compounds–genes	A merged network composed of networks (Figures S1 and S4)				428	652	3.394	0.230	Figure 6
4	*Moringa olifera*–compounds	*Moringa olifera*	1	cpds	18	19	18	1.875	1.0	Figure S2
5	*Moringa olifera*–compounds–genes	*Moringa olifera* compounds	18	Targets	329	330	658	3.380	0.283	Figure S5
6	*Moringa olifera*—compounds–genes	A merged network composed of networks (Figures S2 and S5)				348	673	3.316	0.287	Figure 7
7	*Nigella sativa*–compounds	*Nigella sativa*	1	cpds	15	16	15	1.875	1.0	Figure S3
8	*Nigella sativa*–compounds–genes	*Nigella sativa* compounds	15	Targets	322	347	658	3.380	0.283	Figure S6
9	*Nigella sativa*–compounds–genes	A merged network composed of networks (Figures S3 and S6)				348	673	3.316	0.282	Figure 8

**Table 2:** Binding scores, amino acid interactions, and bond lengths of the selected compounds within the CA-2 active site.

No	Compound	Structure	Binding Scores (kcal/mol^−1^)	Ligand Atom	CA2 Residue	Interaction	Bond Length (A^0^)
1	Nigellidine 1-O-Sulfate	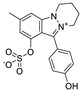	−19.82	Sulfate O	HIS96	HB	2.85
				Sulfate O	THR199	HB	2.45
				Sulfate O	THR199	HB	3.02
				Sulfate O	THR199	HB	2.73
				Zn	HIS94	Ionic	1.97
				Zn	HIS96	Ionic	2.02
				Zn	HIS119	Ionic	2.01
				Sulfate O	Zn	Ionic	2.09
				Sulfate O	Zn	Ionic	2.27
2	Salfredin B_11_	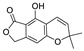	−9.86	Hydroxyl O	THR200	HBD	2.63
				Hydroxyl O	THR200	HBA	2.53
				Zn	HIS94	Ionic	1.97
				Zn	HIS96	Ionic	2.02
				Zn	HIS119	Ionic	2.01
				Carbonyl O	HIS119	Ionic	2.07
3	Ferulic acid	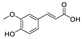	−15.59	Phenolic (OH) COOH (OH) Phenolic OH Methoxy O Methoxy O COOH, OH COOH, CO Zn Zn Zn COOH, OH Benzene ring	ASN67 THR199 ASN62 ASN62 ASN67 THR199 THR199 HIS94 HIS96 HIS119 Zn HIS94	HB HB HB HB HB HB HB Ionic Ionic Ionic Ionic Arene–Arene	1.47 2.51 3.13 2.55 2.81 2.47 2.51 1.97 2.02 2.01 2.06 -
4	Kaempferol	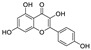	−15.50	2-HO.Ph (OH) 3-HO.Chrom (OH) 2-HO.Ph (OH) Carbonyl O 3-HO.Chrom (OH) Zn Zn Zn 3-HO.Chrom, OH Benzene ring	ASN67 THR199 ASN62 THR199 THR199 HIS94 HIS96 HIS119 Zn HIS94	HB HB HB HB HB Ionic Ionic Ionic Ionic Arene–Arene	1.45 2.56 2.66 2.53 2.56 1.97 2.02 2.01 2.09 -
5	Quercetin	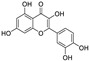	−14.28	2-HO.Ph (OH)	ASN62	HB	2.60
				2-HO.Ph (OH)	ASN62	HB	2.71
				Zn	HIS94	Ionic	1.97
				Zn	HIS96	Ionic	2.02
				Zn	HIS119	Ionic	2.01
				3-HO.Chrom (OH)	Zn	Ionic	2.01
				Carbonyl O	Zn	Ionic	2.53
6	9-(4-Hydroxyphenyl)-2-methoxy-1H-phenalen-1-one	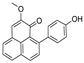	−14.28	Methoxy O Methoxy O Zn Zn Zn Carbonyl O Phenolic benzene ring	THR199(N) THR199(O) HIS94 HIS96 HIS119 Zn HIS94	HB HB Ionic Ionic Ionic Ionic Arene–Arene	2.71 3.03 1.97 2.02 2.01 2.01 -
7	4-Phenyl-1H,3H-naphtho [1,8-cd]pyran-1,3-dione.	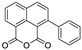	−10.08	Zn	HIS94	Ionic	1.97
				Zn	HIS96	Ionic	2.02
				Zn	HIS119	Ionic	2.01
				Carbonyl C^1^O(O)	Zn	Ionic	2.22
8	Sulfonamide ligand (SUA) inhibitor	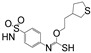	−10.17	O^−^SONH^−^(NH)	THR199	HB	2.89
				O^−^SONH^−^(O)	THR199	HB	2.89
				O^−^SONH^−^(N)	THR199	HB	2.89
				Zn	HIS94	Ionic	1.97
				Zn	HIS96	Ionic	2.02
				Zn	HIS119	Ionic	2.01
				O^−^SONH^−^(O^−^)	Zn	Ionic	2.52
				O^−^SONH^−^(N)	Zn	Ionic	2.31

**Table 3:** Binding scores, amino acid interactions, and bond lengths of the selected compounds within the EGFR active site.

No	Compound	Structure	Binding Scores (kcal/mol^−1^)	Ligand Atom	EGFR Residue	Interaction	Bond Length (A^0^)
1	Nigellidine 1-O-Sulfate	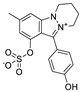	−11.71	4-HO.Ph (OH)	THR766	HB	2.75
				4-HO.Ph (OH)	THR766	HB	2.75
				Sulfate O	MET769	HB	3.17
2	Salfredin B_11_	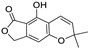	−9.55	Pyran O	H_2_O-THR766	HB	2.75
3	Ferulic acid	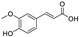	−11.58	Carbonyl O	MET769(O)	HB	2.78
				Phenolic OH	ASP831	HB	1.34
				Methoxy O	H_2_O-THR766	HB	2.52
				Carbonyl O	MET769(N)	HB	3.00
4	Kaempferol	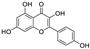	−12.34	3-HO.Chrom (OH)	H_2_O-THR766	HB	2.77
				5-HO.Chrom (OH)	MET769(O)	HB	1.94
				3-HO.Chrom (OH)	H_2_O-THR766	HB	2.77
				Carbonyl O	MET769(N)	HB	2.87
5	Quercetin	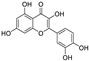	−14.78	2-(3-HO.Ph) OH	MET769(O)	HB	1.45
				5-HO.Chrom (OH)	ASP831	HB	1.24
				Carbonyl O	H_2_O-THR766	HB	2.60
6	9-(4-Hydroxyphenyl)-2-methoxy-1H-phenalen-1-one	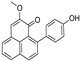	−11.10	Phenolic OH	ASP83	HB	1.30
				Methoxy O	MET769(N)	HB	3.03
7	4-Phenyl-1H,3H-naphtho [1,8-cd]pyran-1,3-dione.	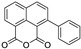	−8.93	Pyran-1-one O	H_2_O-THR766	HB	2.73
8	Erlotinib	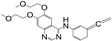	−11.02	Quinazoline N3	H_2_O-THR766	HB	3.02
				Quinazoline N1	MET769	HB	3.09

**Table 4:** Selected compounds with criteria of Lipinski’s rule of five and Veber’s rule.

No	Compound	Structure	Mol. Wt	HBA	HBD	ClogP	No. of Rot.B	TPSA
1	Nigellidine 1-O-Sulfate	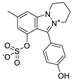	374.417	7	1	2.56	3	158.38
2	Salfredin B_11_	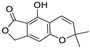	332.235	4	1	2.68	0	126.84
3	Ferulic acid	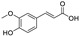	194.186	4	2	1.91	3	140.1
4	Kaempferol	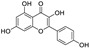	286.239	6	4	2.31	1	38.94
5	Quercetin	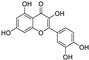	302.238	7	5	2.03	1	38.33
6	9-(4-Hydroxyphenyl)-2-methoxy-1H-phenalen-1-one	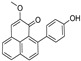	302.329	3	1	5.0	2	110.55
7	4-Phenyl-1H,3H-naphtho [1,8-cd]pyran-1,3-dione.	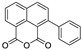	274.275	3	0	4.69	1	102.65

## Data Availability

All data generated or analyzed during this study are included in this published article (and its Supplementary Information files).

## Supplementary Materials

The following supporting information can be downloaded at: https://www.mdpi.com/article/10.3390/ph17060759/s1, Figure S1: Musca accuminata-compounds Network; a network connecting the plant Musca accuminata (in orange rectangle) to the identified compounds (in blue rectangles), Figure S2: Moringa olifera-compounds Network; a network connecting the plant Moringa olifera (in yellow oval shape) to the identified compounds (in pink oval shapes), Figure S3: Nigella sativa-compounds Network; a network connecting the plant Nigella sativa (in yellow circle) to the identified compounds (in blue circles), Figure S4: Musca accuminata compounds-targets; a network links the identified compounds from Musca accuminata (in pink oval shapes) to their target genes (in yellow rectangles), Figure S5: Moringa olifera compounds-targets network: a network describing the identified compounds from Moringa olifera (in blue rectangles) to their target genes (in green rectangles), Figure S6: Nigella compounds-targets network, a merged network illustrates the identified compounds from Nigella sativa (in pink rectangles) to their targets (in violet rectangles), Figure S7: Total ion chromatogram of Nigella Sativa A (Positive mode), B (Negative mode), Figure S8: Total ion chromatogram of Musa acuminata A (positive mode), B (Negative mode), Figure S9: Total ion chromatogram of Moringa olifera A (positive mode), B (Negative mode), Table S1: Metabolomic profiling assisted by HR-LCMS analysis of the crude extracts of Nigella sativa seed, Musca accuminata peels and Moringa olifera seed extracts, Table S2: The top 30 biological KEGG pathways identified by Genes of plant mixture genes related to Human papilloma virus, Table S3: Top 30 biological process identified by plant mixture genes related to Human papilloma virus, Table S4: The top 30 cellular component identified by plant mixture genes related to Human papilloma virus, Table S5: The top 30 molecular functions identified by plant mixture genes related to Human papilloma virus.

## List of Abbreviations

Human papilloma virus: HPV; Middle East respiratory syndrome: MERS; Molecular Operating Environment: MOE; polymer film-forming systems: PFFs; root mean square deviation: RMSD; severe acute respiratory syndrome: SARS.
