# Supplementary material for: In Vitro and Randomized Controlled Clinical Study of Natural Constituents’ Anti-HPV Potential for Treatment of Plantar Warts Supported with In Silico Studies and Network Analysis
Source: Pharmaceuticals (Basel). 2024 Jun 10;17(6):759. doi: 10.3390/ph17060759 (PMC11206833; doi:10.3390/ph17060759)
Supplement: Supplementary file 1 [file pharmaceuticals-17-00759-s001.zip › pharmaceuticals-2993944-supplementary (7).pdf]

<u>**Supplementary file**</u>

In Vitro and Randomized Controlled Clinical Study of Natural Constituents' Anti-HPV Potential for Treatment of Plantar Warts Supported with In Silico Studies and Network Analysis

Nourhan Hisham Shady [1,2,†], Fatma Alzahraa Mokhtar [3,4,†], Hend Samy Abdullah [5], Salah A. Abdel-Aziz [6,7], Soad A. Mohamad [8], Mohamed S. Imam [9,10,*], Sherin Refat El Afify [11] and Usama Ramadan Abdelmohsen [1,12,*]

[1] Department of Pharmacognosy, Faculty of Pharmacy, Deraya University, Universities Zone, New Minia 61111, Egypt; norhan.shady@deraya.edu.eg
[2] Center for Research and Sustainability, Deraya University, Universities Zone, New Minia 61111, Egypt
[3] Fujairah Research Centre, Sakamkam Road, Sakamkam, Fujairah 0000, United Arab Emirates; drfatmaalzahraa1950@gmail.com
[4] Department of Pharmacognosy, Faculty of Pharmacy, El Saleheya El Gadida University, El Saleheya El Gadida, Sharkia 44813, Egypt
[5] Faculty of Pharmacy, Deraya University, Universities Zone, New Minia City 61111, Egypt; hend.samy_1180443@student.deraya.edu.eg
[6] Department of Pharmaceutical Chemistry, Faculty of Pharmacy, Deraya University, Universities Zone, New Minia 61111, Egypt; salah.abdelaziz@deraya.edu.eg
[7] Department of Pharmaceutical Medicinal Chemistry, Faculty of Pharmacy, Al-Azhar University, Assiut 71524, Egypt
[8] Department of Clinical Pharmacy, Faculty of Pharmacy, Deraya University, Universities Zone, New Minia 61111, Egypt; soad.ali@deraya.edu.eg
[9] Department of Clinical Pharmacy, College of Pharmacy, Shaqra University, Shaqra 11961, Saudi Arabia
[10] Department of Clinical Pharmacy, National Cancer Institute, Cairo University, Fom El Khalig Square, Kasr Al-Aini Street, Cairo 11796, Egypt
[11] Department of Pharmacology and Toxicology, Faculty of Pharmacy, Alsalam University, Kafr alzayat, Algharbia 31611, Egypt; sherinelafify@gmail.com
[12] Department of Pharmacognosy, Faculty of Pharmacy, Minia University, Minia 61519, Egypt
* Correspondence: imammohamed311@gmail.com (M.S.I.); usama.ramadan@mu.edu.eg (U.R.A.)
† These authors contributed equally to this work.

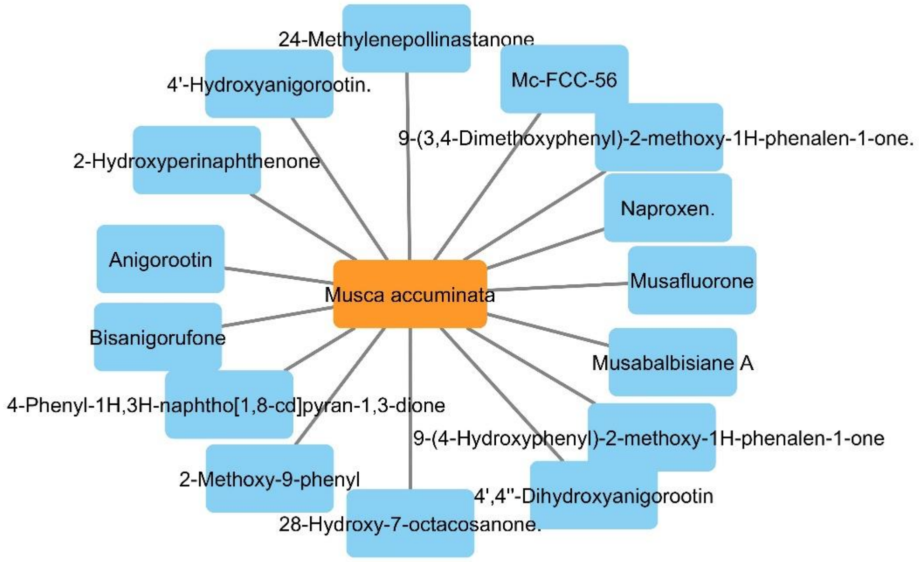

Figure S1: *Musca accuminata*-compounds Network; a network connecting the plant Musca accuminata (in orange rectangle) to the identified compounds (in blue rectangles)

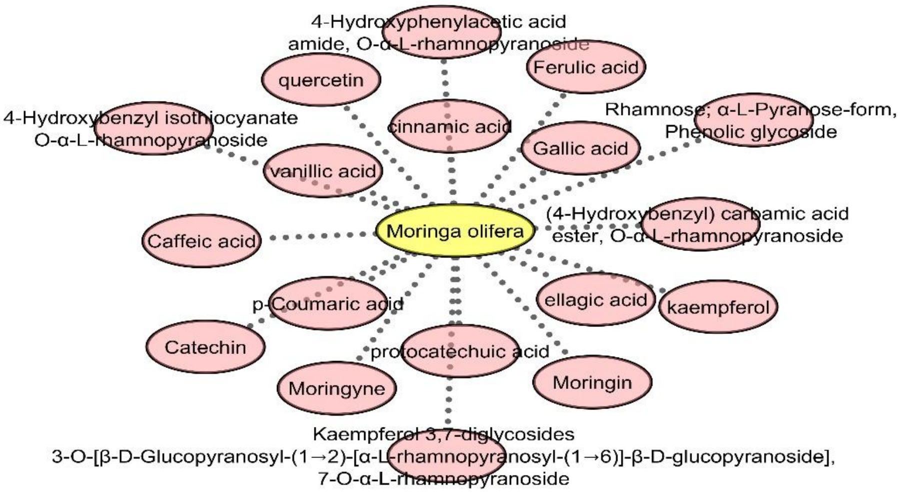

Figure S2: *Moringa olifera*-compounds Network; a network connecting the plant *Moringa olifera* (in yellow oval shape) to the identified compounds (in pink oval shapes)

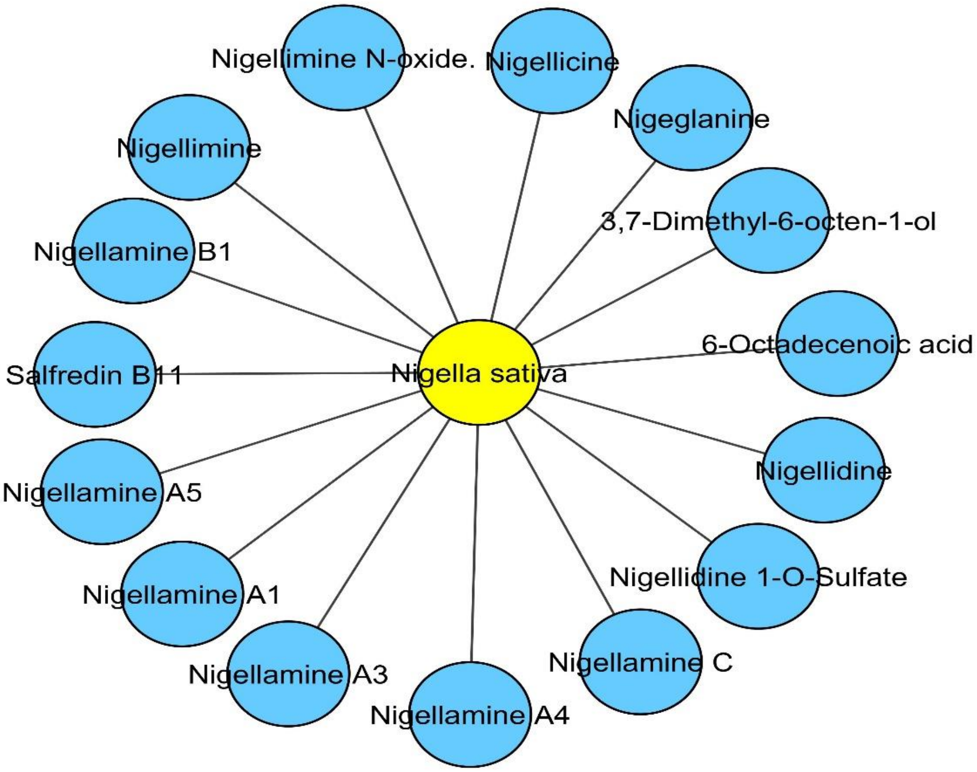

Figure S3: *Nigella sativa*-compounds Network; a network connecting the plant Nigella sativa (in yellow circle) to the identified compounds (in blue circles)

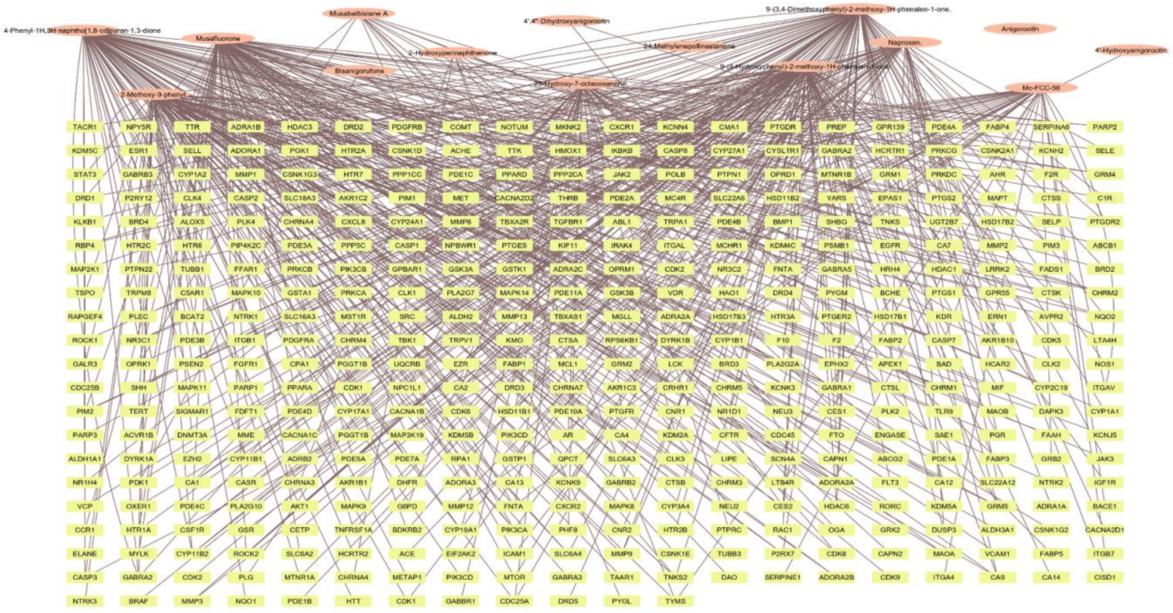

Figure S4: *Musca accuminata* compounds-targets; a network links the identified compounds from *Musca accuminata* (in pink oval shapes) to their target genes (in yellow rectangles)

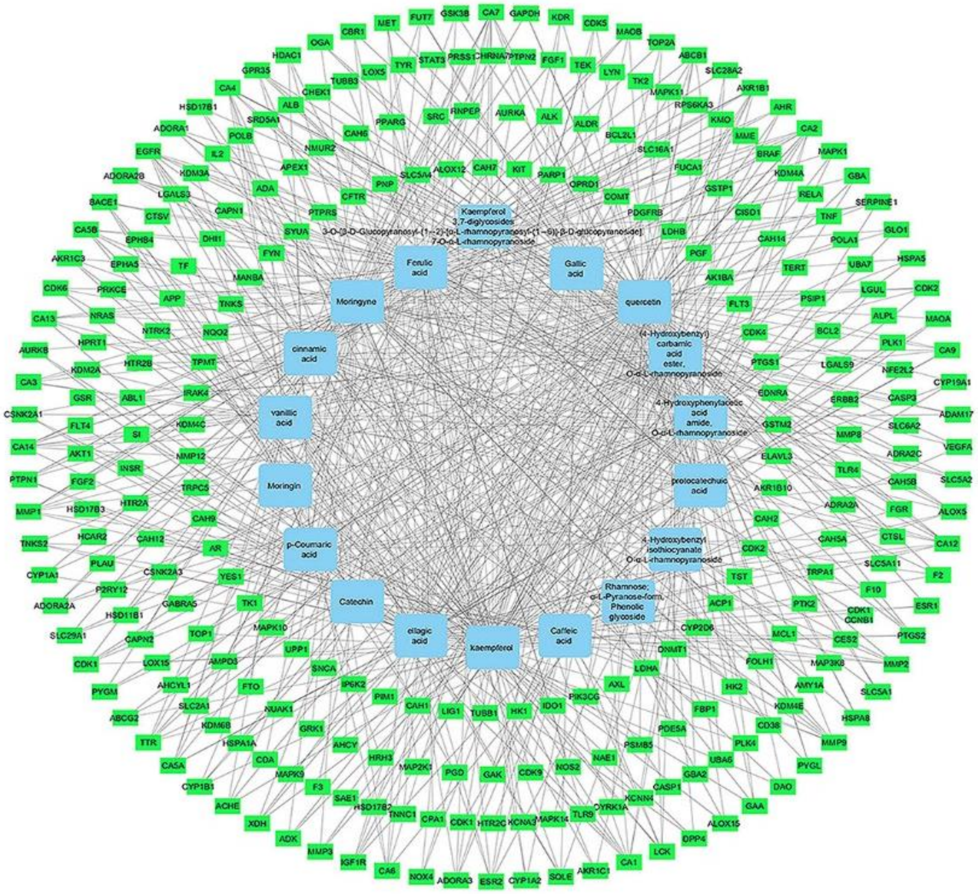

Figure S5: *Moringa olifera* compounds-targets network: a network describing the identified compounds from *Moringa olifera* (in blue rectangles) to their target genes (in green rectangles)

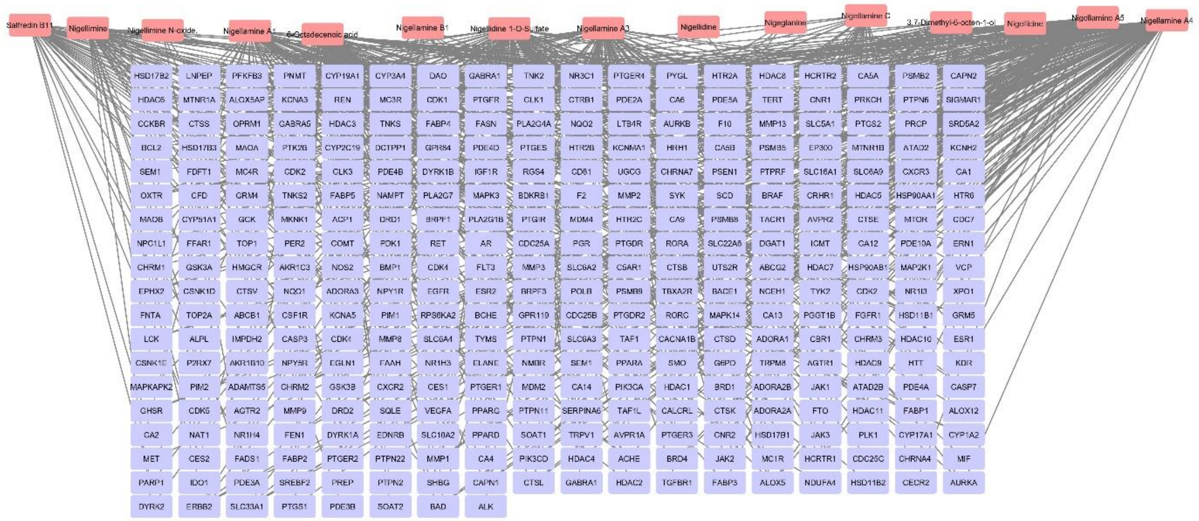

Figure S6: Nigella compounds-targets network, a merged network illustrates the identified compounds from *Nigella sativa* (in pink rectangles) to their targets (in violet rectangles)

Table S1: LC-MS Assisted Dereplication of the Chemical Constituents in Nigella sativa seed, Musca accuminata peels and Moringa olifera seed extracts.

No	Name	structure	Exact mass	Molecular formula	Reference
1	Nigellimine	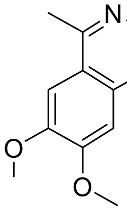	203.094629	$C_{12}H_{13}NO_2$	[1]
2	Nigellimine N-oxide.	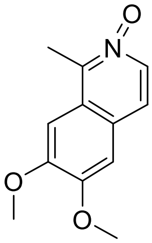	219.089544	$C_{12}H_{13}NO_3$	[2]
3	3,7-Dimethyl-6-octen-1-ol.	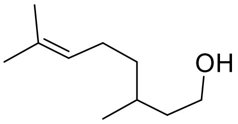	156.151415	$C_{10}H_2O$	[3]
4	Nigeglanine	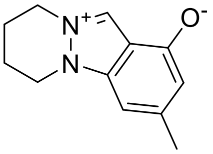	202.110613	$C_{12}H_{14}NO$	[4]
5	Salfredin B_{11}	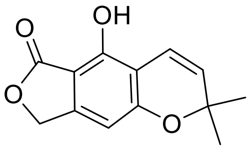	232.07356	$C_{13}H_{12}O_4$	[5]
6	Nigellicine	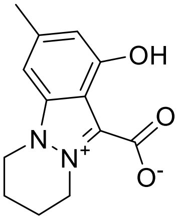	246.100443	$C_{13}H_{14}N_2O_3$	[6]

7	6-Octadecenoic acid	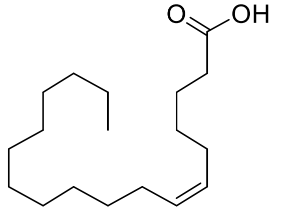	282.25588	$C_{18}H_{34}O_2$	[7]
8	Nigellidine	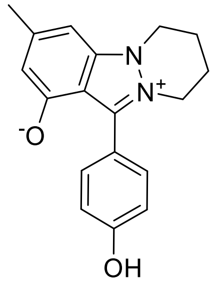	294.136828	$C_{18}H_{18}N_2O_2$	[8]
9	Nigellidine 1-O-Sulfate	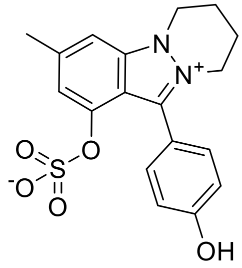	374.093643	$C_{18}H_{18}N_2O_5S$	[9]
10	Nigellamine C.	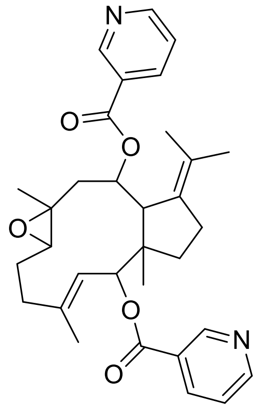	530.27807	$C_{32}H_{38}N_2O_5$	[10]
111	Nigellamine A₄.	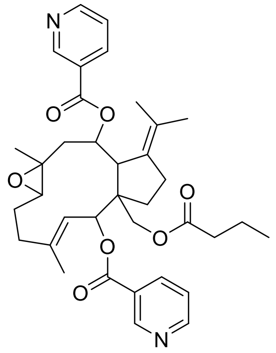	616.31485	$C_{36}H_{44}N_2O_7$	[10]

12	Nigellamine A_3.	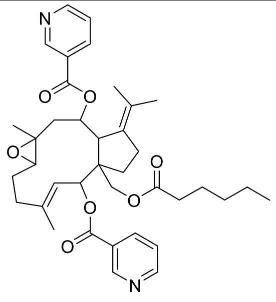	644.346153	$C_{38}H_{48}N_2O_7$	[10]
13	Nigellamine A_1.	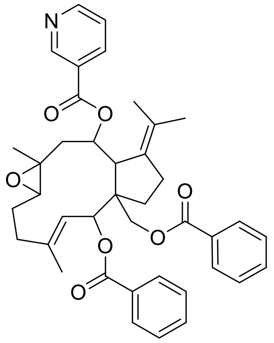	649.303954	$C_{40}H_{43}NO_7$	[11]
14	Nigellamine A_5.	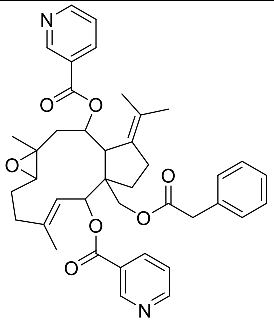	664.314853	$C_{40}H_{44}N_2O_7$	[10]
15	Nigellamine B1.	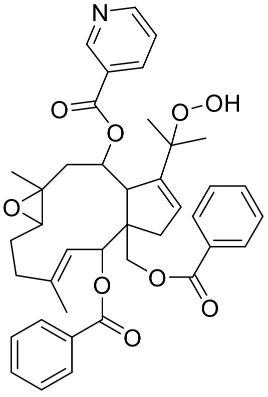	681.293784	$C_{40}H_{43}NO_9$	[11]

16	28-Hydroxy-7-octacosanone.	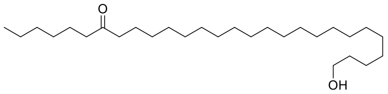	424.428	$C_{28}H_{56}O_2$	[12]
17	Musabalbisiane A	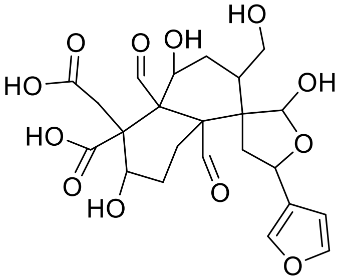	498.172	$C_{23}H_{30}O_{12}$	[13]
18	2-Hydroxyperinaphthenone.	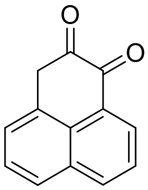	196.052	$C_{13}H_8O_2$	[14]
19	Naproxen.	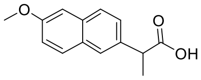	230.094	$C_{14}H_{14}O_3$	[15]
20	4-Phenyl-1H,3H-naphtho[1,8-cd]pyran-1,3-dione.	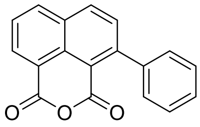	274.062	$C_{18}H_{10}O_3$	[14]
21	2-Methoxy-9-phenyl-1H-phenalen-1-one.	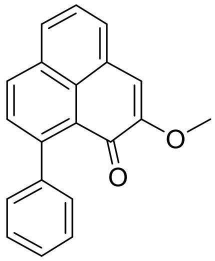	286.099	$C_{20}H_{14}O_2$	[14]
22	Musafluorone	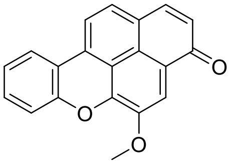	300.078	$C_{20}H_{12}O_3$	[16]

23	9-(4-Hydroxyphenyl)-2-methoxy-1H-phenalen-1-one	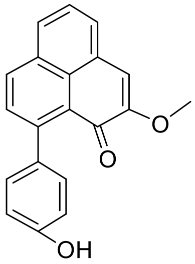	302.094	$C_{20}H_{14}O_3$	[14]
24	9-(3,4-Dimethoxyphenyl)-2-methoxy-1H-phenalen-1-one.	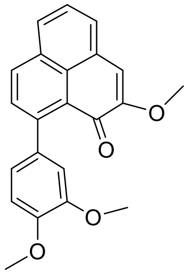	346.120	$C_{22}H_{18}O_4$	[17]
25	Bisanigorufone	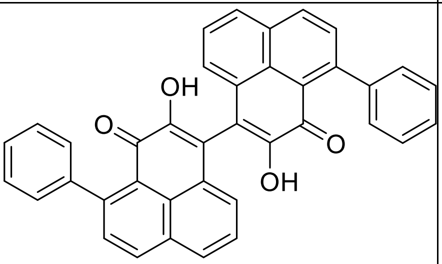	542.151	$C_{38}H_{22}O_4$	[18]
26	Anigorootin	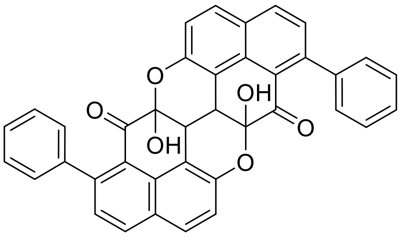	574.141 64	$C_{38}H_{22}O_6$	[19]
27	4'-Hydroxyanigorootin.	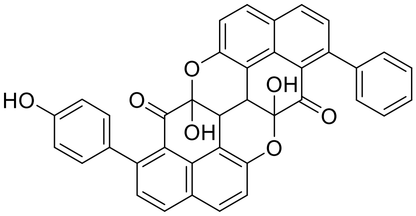	590.136 555	$C_{38}H_{22}O_7$	[20]
28	4',4''-Dihydroxyanigorootin.	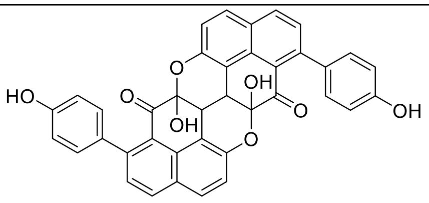	606.131 47	$C_{38}H_{22}O_8$	[20]

29	Mc-FCC-56. Musa cavendish fluorescent chlorophyll catabolite	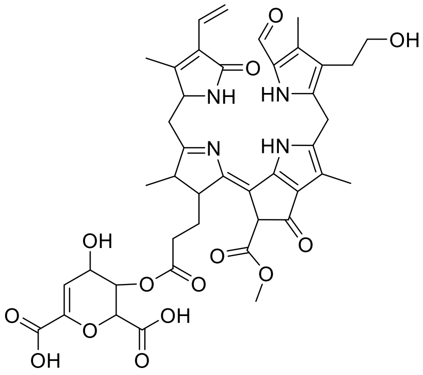	830.301056	$C_{42}H_{46}N_4O_{14}$	[21]
30	24-Methylenepollinastanone	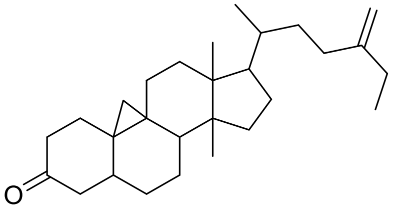	410.354865	$C_{29}H_{46}O$	[22]
31	Moringyne	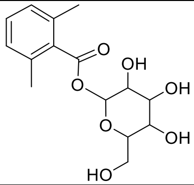	312.12090	$C_{15}H_{20}O_7$	[23]
32	Catechin	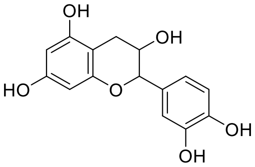	290.07904	$C_{15}H_{14}O_6$	[24]
33	Quercetin	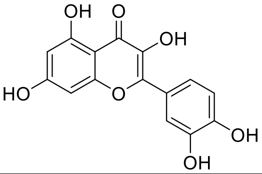	302.042655	$C_{15}H_{10}O_7$	[24]
34	Kaempferol	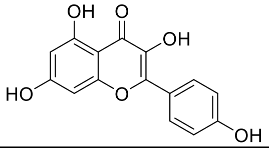	286.04774	$C_{15}H_{10}O_6$	[25]
35	Gallic acid	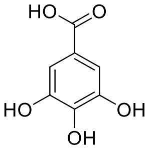	170.021525	$C_7H_6O_5$	[26]
36	*p*-Coumaric acid	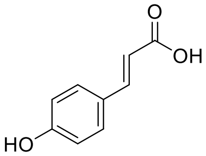	164.047345	$C_9H_8O_3$	[24]

37	Ferulic acid	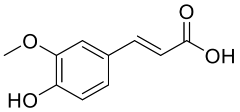	194.058	$C_{10}H_{10}O_4$	[24]
38	Caffeic acid	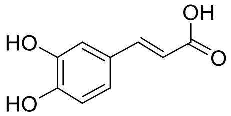	180.04226	$C_9H_8O_4$	[24]
39	Protocatechuic acid	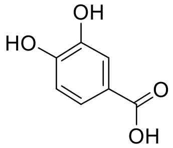	154.02661	$C_7H_6O_4$	[24]
40	Cinnamic acid	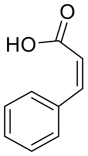	148.05243	$C_9H_8O_2$	[24]
41	Ellagic acid	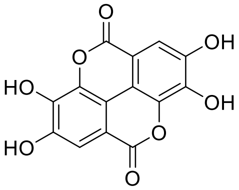	302.00627	$C_{14}H_6O_8$	[25]
42	Vanillic acid	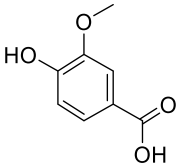	168.04226	$C_8H_8O_4$	[24]
43	Moringine	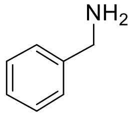	107.073499	C_7H_9N	[27]
44	Kaempferol 3,7-diglycosides 3-O-[β-D-Glucopyranosyl-(1→2)-[α-L-rhamnopyranosyl-(1→6)]-β-D-glucopyranoside], 7-O-α-L-rhamnopyranoside	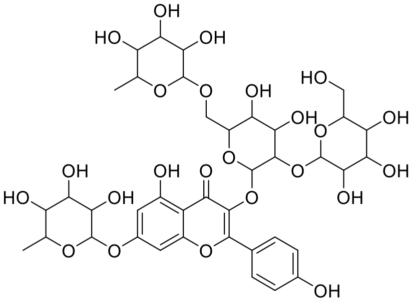	902.26921	$C_{39}H_{50}O_{24}$	[28]
45	4-Hydroxybenzyl isothiocyanate *O*-α-L-rhamnopyranoside	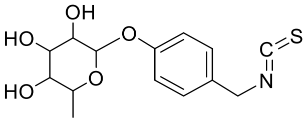	311.082744	$C_{14}H_{17}NO_5S$	[29]

46	(4-Hydroxybenzyl) carbamic acid ester, *O*-α-L-rhamnopyranoside	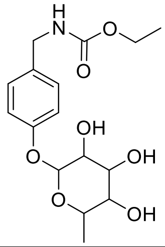	341.147 454	$C_{16}H_{23}NO_7$	[30]
47	Rhamnose; α-L-Pyranose-form, Phenolic glycoside	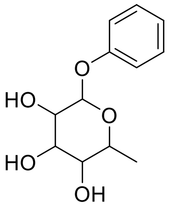	240.099 7	$C_{12}H_{16}O_5$	[31]
48	4-Hydroxyphenylacetic acid amide, *O*-α-L-rhamnopyranoside	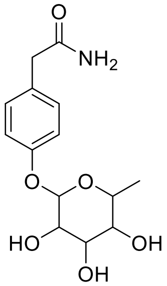	297.121 239	$C_{14}H_{19}NO_6$	[31]
49	3,4-Dihydro-4,8-dihydroxy-3-methyl-1H-2-benzopyran-1-one; (3*R*,4*S*)-form	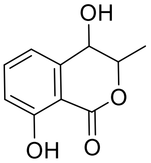	194.057 91	$C_{10}H_{10}O_4$	[32]

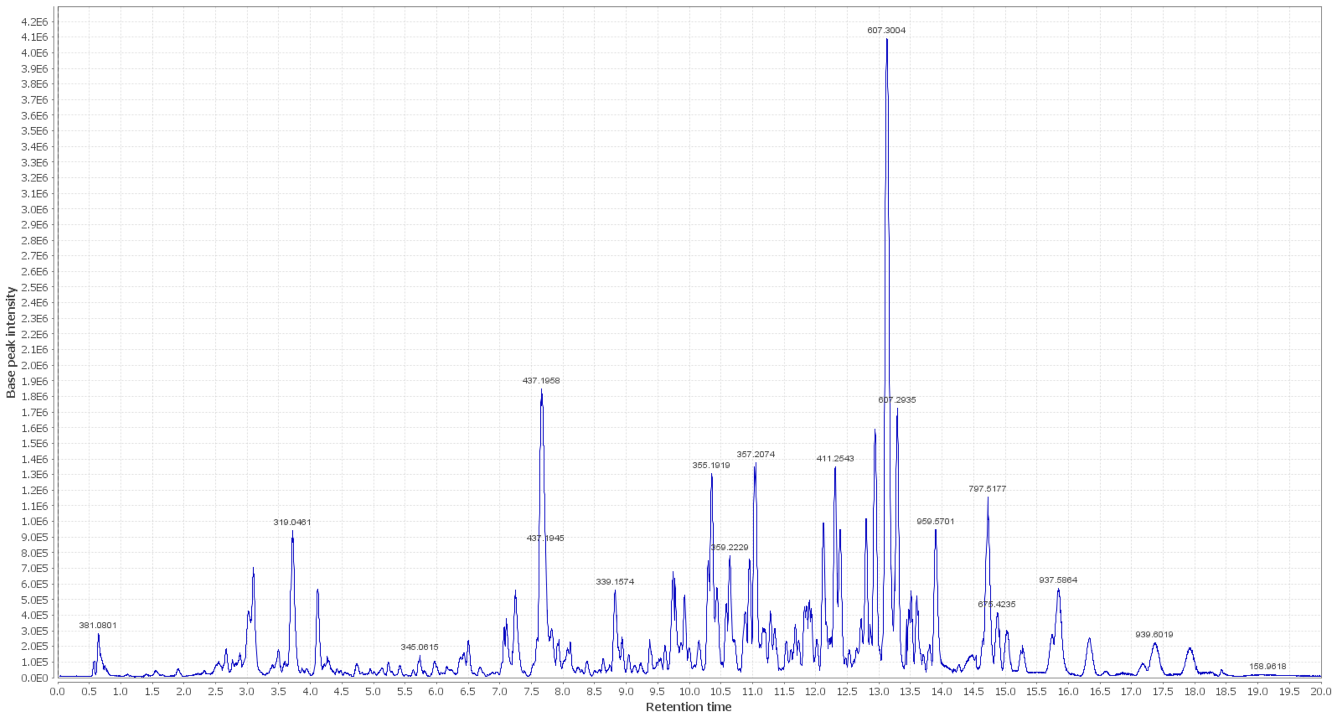

A

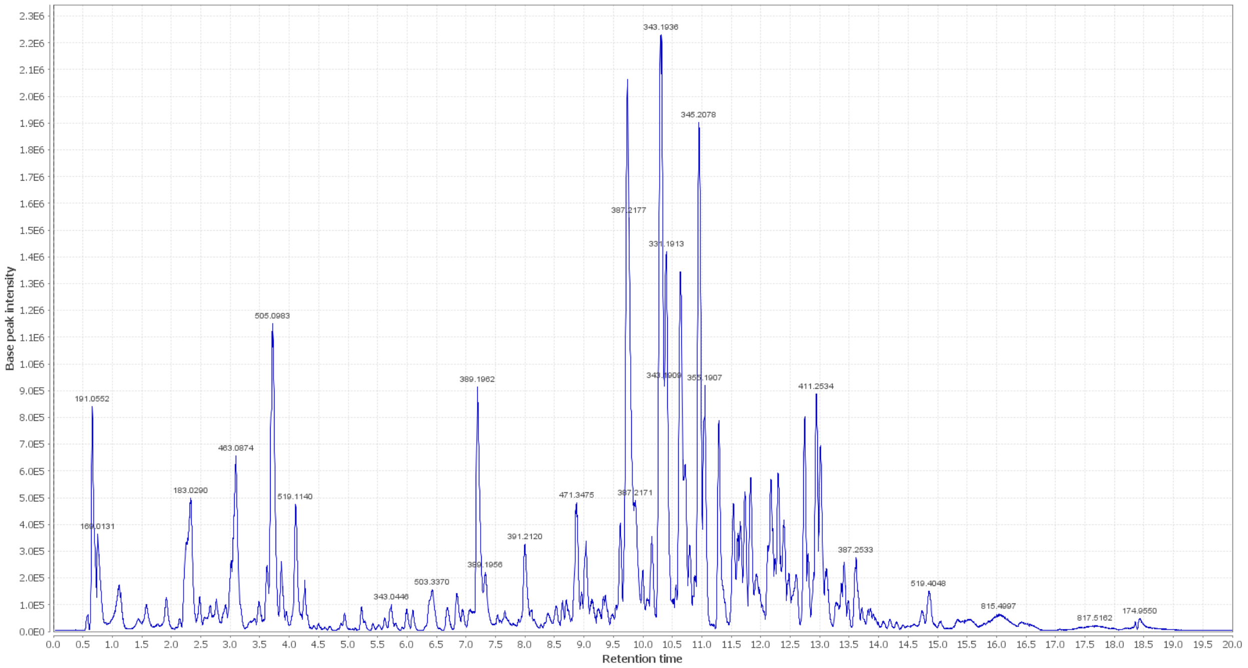

B

Figure S7: Total ion chromatogram of *Nigella Sativa* A (Positive mode), B (Negative mode).

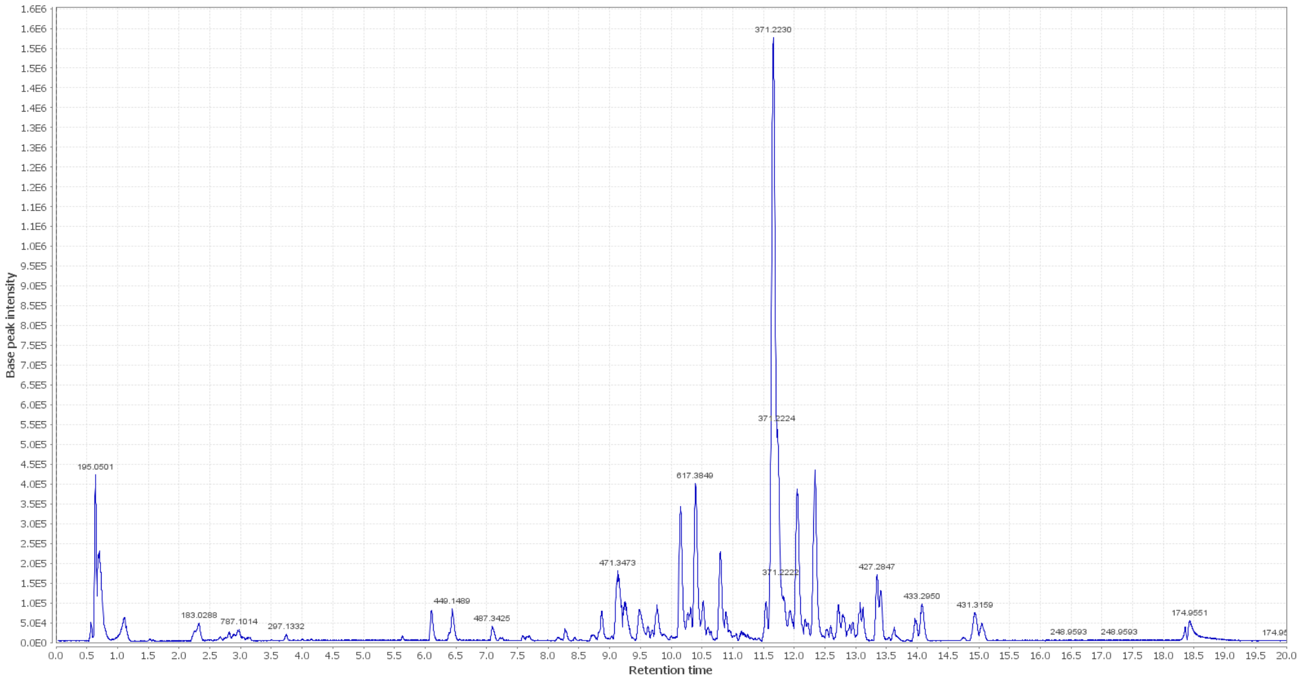

A

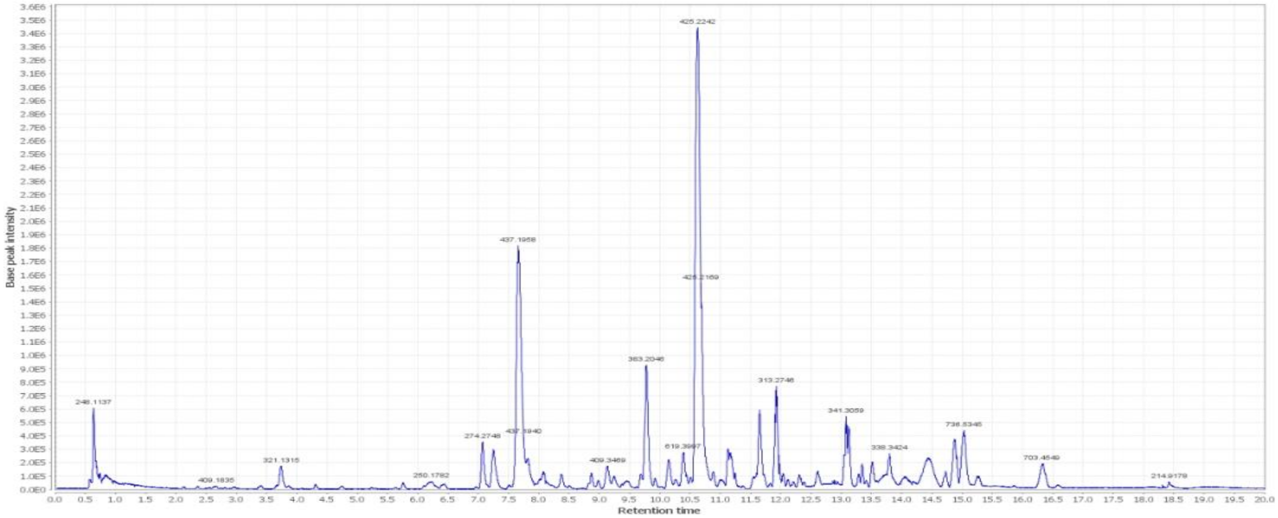

B

Figure S8: Total ion chromatogram of *Musa acuminata* A (positive mode), B (Negative mode).

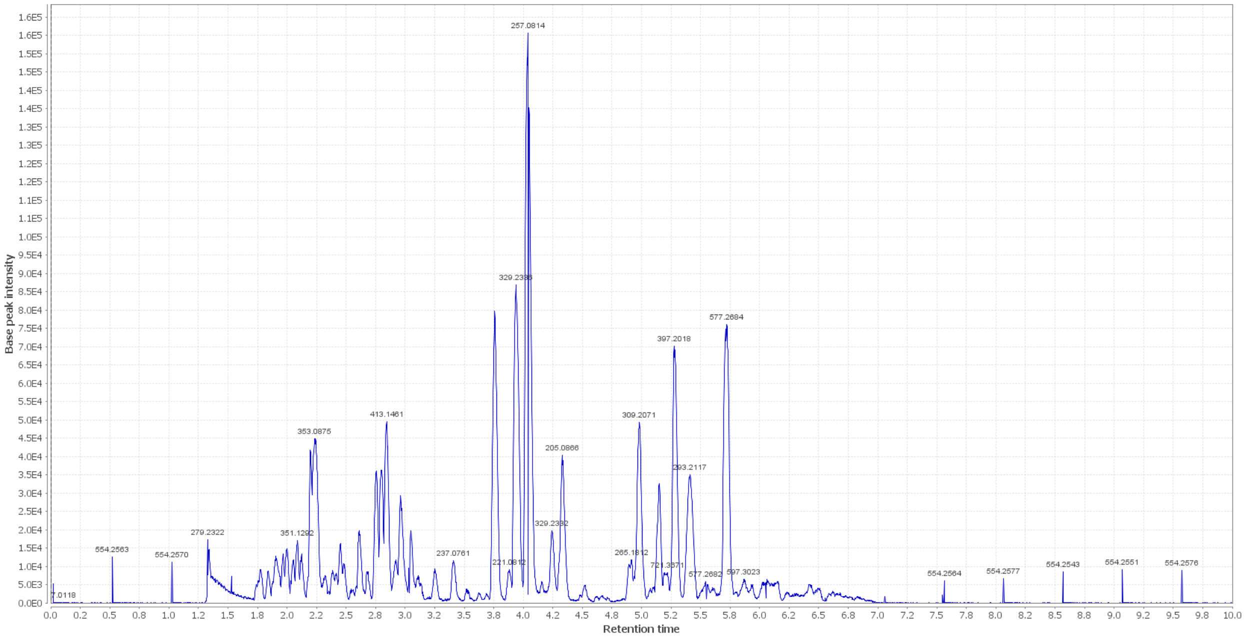

A

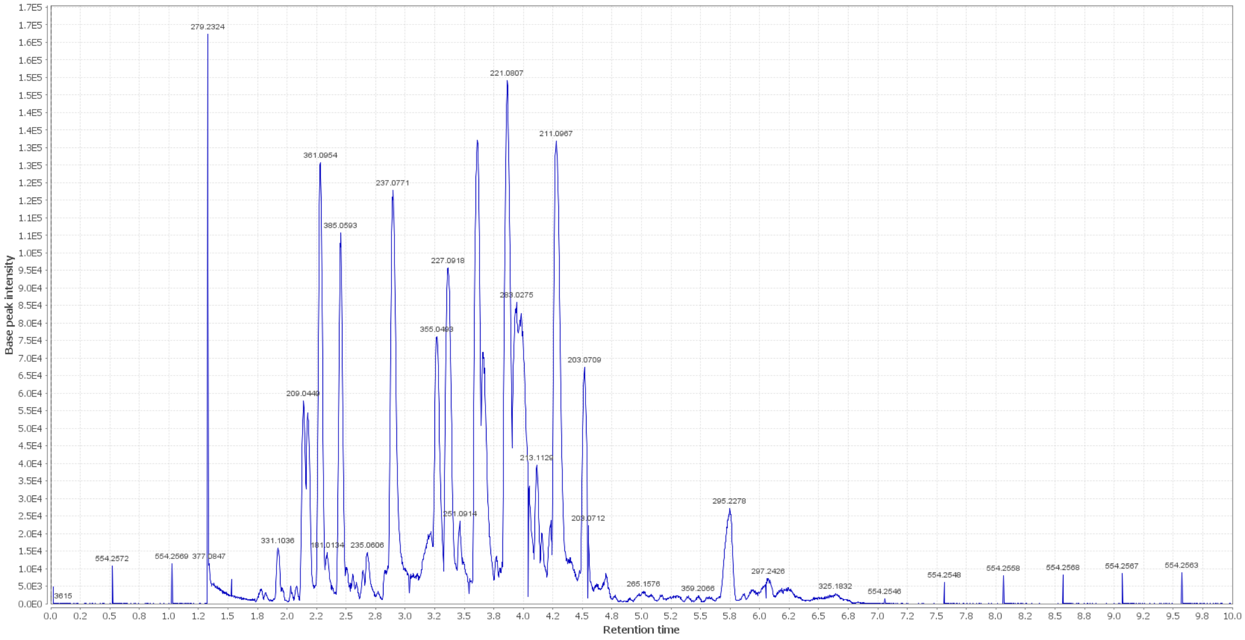

B

Figure S9: Total ion chromatogram of *Moringa Olifera* A (positive mode), B (Negative mode).

Table S2: The top 30 biological KEGG pathways identified by Genes of plant mixture genes related to Human papilloma virus.

NO	Pathway	Fold Enrichment	nGenes	Genes
1	Positive regulation of mast cell chemotaxis	345.39	2	VEGFA PGF
2	Pyrimidine deoxyribonucleoside monophosphate biosynthetic process	345.39	1	TYMS
3	Peptidyl-serine ADP-ribosylation	345.39	1	PARP1
4	Uracil metabolic process	345.39	1	TYMS
5	DNA ADP-ribosylation	345.39	1	PARP1
6	VEGF-activated platelet-derived growth factor receptor signaling pathway	345.39	1	VEGFA
7	Positive regulation of cell proliferation by VEGF-activated platelet derived growth factor receptor signaling pathway	345.39	1	VEGFA
8	Prostate epithelial cord elongation	345.39	1	ESR1
9	Tertiary branching involved in mammary gland duct morphogenesis	345.39	1	PGR
10	Cardiac vascular smooth muscle cell development	345.39	1	VEGFA
11	Platelet-derived growth factor production	345.39	1	PTGS2
12	Regulation of platelet-derived growth factor production	345.39	1	PTGS2
13	Regulation of G2/MI transition of meiotic cell cycle	345.39	1	CDC25A
14	Positive regulation of G2/MI transition of meiotic cell cycle	345.39	1	CDC25A
15	Regulation of RNA interference	345.39	1	TERT
16	Positive regulation of axon guidance	345.39	1	VEGFA
17	Positive regulation of prolactin secretion	345.39	1	EGFR
18	Negative regulation of adherens junction organization	345.39	1	VEGFA
19	Regulation of single strand break repair	345.39	1	PARP1
20	Response to hydrogen sulfide	345.39	1	NQO1

21	Negative regulation of blood-brain barrier permeability	345.39	1	VEGFA
22	Negative regulation of steroid hormone secretion	345.39	1	PTPN11
23	Negative regulation of corticosteroid hormone secretion	345.39	1	PTPN11
24	Negative regulation of glucocorticoid secretion	345.39	1	PTPN11
25	Negative regulation of ATP biosynthetic process	345.39	1	PARP1
26	Mammary gland branching involved in pregnancy	296.05	2	PGR ESR1
27	Regulation of mast cell chemotaxis	259.05	2	VEGFA PGF
28	DTTP biosynthetic process	259.05	1	TYMS
29	G2/MI transition of meiotic cell cycle	259.05	1	CDC25A
30	Pyrimidine deoxyribonucleoside triphosphate biosynthetic process	259.05	1	TYMS

Table S3: top 30 biological process identified by plant mixture genes related to Human papilloma virus

NO	Pathway	Fold Enrichment	nGenes	Genes
1	Positive regulation of mast cell chemotaxis	345.39	2	VEGFA PGF
2	Pyrimidine deoxyribonucleoside monophosphate biosynthetic process	345.39	1	TYMS
3	Peptidyl-serine ADP-ribosylation	345.39	1	PARP1
4	Uracil metabolic process	345.39	1	TYMS
5	DNA ADP-ribosylation	345.39	1	PARP1
6	VEGF-activated platelet-derived growth factor receptor signaling pathway	345.39	1	VEGFA
7	Positive regulation of cell proliferation by VEGF-activated platelet derived growth factor receptor signaling pathway	345.39	1	VEGFA
8	Prostate epithelial cord elongation	345.39	1	ESR1
9	Tertiary branching involved in mammary gland duct morphogenesis	345.39	1	PGR
10	Cardiac vascular smooth muscle cell development	345.39	1	VEGFA
11	Platelet-derived growth factor production	345.39	1	PTGS2
12	Regulation of platelet-derived growth factor production	345.39	1	PTGS2
13	Regulation of G2/MI transition of meiotic cell cycle	345.39	1	CDC25A
14	Positive regulation of G2/MI transition of meiotic cell cycle	345.39	1	CDC25A
15	Regulation of RNA interference	345.39	1	TERT
16	Positive regulation of axon guidance	345.39	1	VEGFA
17	Positive regulation of prolactin secretion	345.39	1	EGFR
18	Negative regulation of adherens junction organization	345.39	1	VEGFA
19	Regulation of single strand break repair	345.39	1	PARP1
20	Response to hydrogen sulfide	345.39	1	NQO1
21	Negative regulation of blood-brain barrier permeability	345.39	1	VEGFA

22	Negative regulation of steroid hormone secretion	345.39	1	PTPN11
23	Negative regulation of corticosteroid hormone secretion	345.39	1	PTPN11
24	Negative regulation of glucocorticoid secretion	345.39	1	PTPN11
25	Negative regulation of ATP biosynthetic process	345.39	1	PARP1
26	Mammary gland branching involved in pregnancy	296.05	2	PGR ESR1
27	Regulation of mast cell chemotaxis	259.05	2	VEGFA PGF
28	DTTP biosynthetic process	259.05	1	TYMS
29	G2/MI transition of meiotic cell cycle	259.05	1	CDC25A
30	Pyrimidine deoxyribonucleoside triphosphate biosynthetic process	259.05	1	TYMS

Table S4: The top 30 cellular component identified by plant mixture genes related to Human papilloma virus

NO	Pathway	Fold Enrichment	nGenes	Genes
1	RNA-directed RNA polymerase complex	345.39	1	TERT
2	Cyclin D2-CDK4 complex	345.39	1	CDK4
3	Multivesicular body, internal vesicle	259.05	1	EGFR
4	Alpha-beta T cell receptor complex	207.24	1	PTPN6
5	Sorting endosome	172.70	1	PTPN1
6	Mitochondrial crista	129.52	1	PTPN1
7	Transcription preinitiation complex	103.62	1	ESR1
8	NMDA selective glutamate receptor complex	94.20	1	PTK2B
9	Telomere cap complex	74.01	1	TERT
10	Nuclear telomere cap complex	74.01	1	TERT
11	Chromosome, telomeric repeat region	74.01	1	TERT
12	Cytoplasmic side of endoplasmic reticulum membrane	64.76	1	PTPN1
13	T cell receptor complex	60.95	1	PTPN6
14	Apical dendrite	57.57	1	PTK2B
15	Telomerase holoenzyme complex	49.34	1	TERT
16	Cyclin-dependent protein kinase holoenzyme complex	47.10	2	CDK4 CDK1
17	Transcriptionally active chromatin	43.17	1	ESR1
18	Nuclear outer membrane	35.73	1	PTGS2
19	Microvillus membrane	34.54	1	CA9
20	Tertiary granule lumen	30.93	2	MMP9 PTPN6
21	Mediator complex	23.03	1	CDK4
22	Microvillus	22.28	2	CA2 CA9
23	Ionotropic glutamate receptor complex	22.05	1	PTK2B
24	Nucleoid	22.05	1	TERT
25	Mitochondrial nucleoid	22.05	1	TERT
26	Neurotransmitter receptor complex	21.15	1	PTK2B
27	Serine/threonine protein kinase complex	19.93	2	CDK4 CDK1
28	Chromosome, telomeric region	17.37	3	PARP1 TERT CDK1
29	Protein kinase complex	17.13	2	CDK4 CDK1
30	Plasma membrane raft	16.58	2	PTGS2 PTPN11

Table S5: The top 30 molecular functions identified by plant mixture genes related to Human papilloma virus

NO	nGenes	Fold Enrichment	Pathway	Genes
1	1	345.39	NADPH dehydrogenase (quinone) activity	NQO1
2	1	345.39	Estrogen response element binding	ESR1
3	1	345.39	Vascular endothelial growth factor receptor 1 binding	VEGFA
4	1	345.39	NAD DNA ADP-ribosyltransferase activity	PARP1
5	1	259.05	TFIIB-class transcription factor binding	ESR1
6	1	259.05	Epidermal growth factor binding	EGFR
7	1	259.05	Receptor-receptor interaction	FGFR1
8	2	230.26	Nitric-oxide synthase regulator activity	ESR1 EGFR
9	1	207.24	Superoxide dismutase activity	NQO1
10	1	207.24	Fibroblast growth factor-activated receptor activity	FGFR1
11	1	207.24	Oxidoreductase activity, acting on superoxide radicals as acceptor	NQO1
12	1	207.24	Estrogen receptor activity	ESR1
13	1	207.24	Neuropilin binding	VEGFA
14	1	172.70	Telomerase activity	TERT
15	1	172.70	RNA-directed DNA polymerase activity	TERT
16	1	172.70	Arylesterase activity	CA2
17	1	172.70	Phosphorylation-dependent protein binding	PTPN6
18	2	159.41	RNA polymerase II CTD heptapeptide repeat kinase activity	CDK4 CDK1
19	1	148.03	Cytochrome-b5 reductase activity, acting on NAD(P)H	NQO1
20	2	138.16	Carbonate dehydratase activity	CA2 CA9
21	2	129.52	Vascular endothelial growth factor receptor binding	VEGFA PGF
22	1	129.52	NMDA glutamate receptor activity	PTK2B
23	1	115.13	Non-membrane spanning protein tyrosine phosphatase activity	PTPN11
24	1	115.13	D1 dopamine receptor binding	PTPN11
25	2	103.62	Transcription coactivator binding	ESR1 TERT

26	1	103.62	Oxidoreductase activity, acting on NAD(P)H, heme protein as acceptor	NQO1
27	1	94.20	Vascular endothelial growth factor receptor 2 binding	VEGFA
28	1	94.20	Insulin receptor substrate binding	PTPN11
29	2	90.10	Insulin receptor binding	PTPN11<br>PTPN1
30	1	86.35	Sequence-specific mRNA binding	TYMS

1. Malik, S.; Zaman, K. J. J. o. N. P., Nigellimine: a new isoquinoline alkaloid from the seeds of Nigella sativa. **1992,** 55, (5), 676-678.
2. MALIK, S.; AHMAD, S.; CHAUDHARY, I. J. H., Nigellimine N-oxide-a new isoquinoline alkaloid from the seeds of Nigella sativa. **1985,** 23, (4), 953-955.
3. Rajendran, J.; Pachaiappan, P.; Thangarasu, R. J. N.; Cancer, Citronellol, an acyclic monoterpene induces mitochondrial-mediated apoptosis through activation of proapoptotic factors in MCF-7 and MDA-MB-231 human mammary tumor cells. **2021,** 73, (8), 1448-1458.
4. Liu, Y.-M.; Yang, J.-S.; Liu, Q.-H. J. C.; bulletin, p., A new alkaloid and its artificial derivative with an indazole ring from Nigella glandulifera. **2004,** 52, (4), 454-455.
5. Liu, X.; Aisa, H. A.; Xin, X. J. N. p. r., A new fatty acid ester from Nigella sativa var. hispidula Boiss showing potent anti-protein tyrosine phosphatase 1B activity. **2019,** 33, (4), 472-476.
6. Malik, S.; Cun-Heng, H.; Clardy, J. J. T. l., Isolation and structure determination of nigellicine, a novel alkaloid from the seeds of Nigella sativa. **1985,** 26, (23), 2759-2762.
7. Khairalla, M. E. K. Extraction, Constituent and Biological Activity of some Phytochemicals. Sudan University of Science and Technology, 2020.
8. Malik, S.; Hasan, S. S.; Choudhary, M. I.; Ni, C.-Z.; Clardy, J. J. T. l., Nigellidine—a new indazole alkaloid from the seeds of Nigella sativa. **1995,** 36, (12), 1993-1996.
9. Ali, Z.; Ferreira, D.; Carvalho, P.; Avery, M. A.; Khan, I. A., Nigellidine-4-O-sulfite, the first sulfated indazole-type alkaloid from the seeds of Nigella sativa. *J Nat Prod* **2008,** 71, (6), 1111-2.
10. Morikawa, T.; Xu, F.; Ninomiya, K.; Matsuda, H.; Yoshikawa, M., Nigellamines A3, A4, A5, and C, new dolabellane-type diterpene alkaloids, with lipid metabolism-promoting activities from the Egyptian medicinal food black cumin. *Chemical & pharmaceutical bulletin* **2004,** 52, (4), 494-7.
11. Morikawa, T.; Xu, F.; Kashima, Y.; Matsuda, H.; Ninomiya, K.; Yoshikawa, M., Novel Dolabellane-Type Diterpene Alkaloids with Lipid Metabolism Promoting Activities from the Seeds of Nigella sativa. *Organic Letters* **2004,** 6, (6), 869-872.
12. Vilela, C.; Santos, S. A.; Villaverde, J. J.; Oliveira, L.; Nunes, A.; Cordeiro, N.; Freire, C. S.; Silvestre, A. J., Lipophilic phytochemicals from banana fruits of several Musa species. *Food Chem* **2014,** 162, 247-52.
13. Ali, M. J. P., Neo-clerodane diterpenoids from Musa balbisiana seeds. **1992,** 31, (6), 2173-2175.
14. Otálvaro, F.; Nanclares, J.; Vásquez, L. E.; Quinones, W.; Echeverri, F.; Arango, R.; Schneider, B. J. J. o. n. p., Phenalenone-type compounds from Musa acuminata var."Yangambi km 5"(AAA) and their activity against Mycosphaerella fijiensis. **2007,** 70, (5), 887-890.
15. Abad, T.; McNaughton-Smith, G.; Fletcher, W. Q.; Echeverri, F.; Diaz-Peñate, R.; Tabraue, C.; Ruiz de Galarreta, C. M.; López-Blanco, F.; Luis, J. G., Isolation of (S)-(+)-naproxene from Musa acuminata. Inhibitory effect of naproxene and its 7-methoxy isomer on constitutive COX-1 and inducible COX-2. *Planta Med* **2000,** 66, (5), 471-3.

16. Duque, L.; Restrepo, C.; Sáez, J.; Gil, J.; Schneider, B.; Otálvaro, F. J. T. L., Synthesis of musafluorone: a naphthoxanthenone isolated from Musa acuminata. **2010,** 51, (35), 4640-4643.
17. Quiñones, W.; Escobar, G.; Echeverri, F.; Torres, F.; Rosero, Y.; Arango, V.; Cardona, G.; Gallego, A., Synthesis and Antifungal Activity of Musa Phytoalexins and Structural Analogs. **2000,** 5, (7), 974-980.
18. Yannai, S., *Dictionary of food compounds with CD-ROM*. Crc Press: 2012.
19. Otálvaro, F.; Görls, H.; Hölscher, D.; Schmitt, B.; Echeverri, F.; Quiñones, W.; Schneider, B., Dimeric phenylphenalenones from Musa acuminata and various Haemodoraceae species. Crystal structure of anigorootin. *Phytochemistry* **2002,** 60, (1), 61-6.
20. Jitsaeng, K.; Paetz, C.; Schneider, B. J. R. o. N. P., Phenylphenalenones from Musa cv.'Thepanom'(BBB). **2010,** 4, (1), 26-30.
21. Moser, S.; Müller, T.; Ebert, M. O.; Jockusch, S.; Turro, N. J.; Kräutler, B. J. A. C. I. E., Blue luminescence of ripening bananas. **2008,** 47, (46), 8954-8957.
22. Koorbanally, N.; Mulholland, D. A.; Crouch, N., Alkaloids and triterpenoids from Ammocharis coranica (Amaryllidaceae). *Phytochemistry* **2000,** 54, (1), 93-97.
23. Memon, G. M.; Memon, S. A.; Memon, A. R., Isolation and structure elucidation of moringyne - a new glycoside from seeds of Moringa oleifera Lam. *Pakistan journal of scientific and industrial research* **1985**.
24. Govardhan Singh, R. S.; Negi, P. S.; Radha, C., Phenolic composition, antioxidant and antimicrobial activities of free and bound phenolic extracts of Moringa oleifera seed flour. *Journal of Functional Foods* **2013,** 5, (4), 1883-1891.
25. Singh, B. N.; Singh, B. R.; Singh, R. L.; Prakash, D.; Dhakarey, R.; Upadhyay, G.; Singh, H. B., Oxidative DNA damage protective activity, antioxidant and anti-quorum sensing potentials of Moringa oleifera. *Food and chemical toxicology : an international journal published for the British Industrial Biological Research Association* **2009,** 47, (6), 1109-16.
26. Zhu, Y.; Yin, Q.; Yang, Y., Comprehensive Investigation of Moringa oleifera from Different Regions by Simultaneous Determination of 11 Polyphenols Using UPLC-ESI-MS/MS. *Molecules* **2020,** 25, (3).
27. Cirmi, S.; Ferlazzo, N.; Gugliandolo, A.; Musumeci, L.; Mazzon, E.; Bramanti, A.; Navarra, M., Moringin from Moringa Oleifera Seeds Inhibits Growth, Arrests Cell-Cycle, and Induces Apoptosis of SH-SY5Y Human Neuroblastoma Cells through the Modulation of NF-κB and Apoptotic Related Factors. *International journal of molecular sciences* **2019,** 20, (8).
28. Marzouk, M. M.; Al-Nowaihi, A.-S. M.; Kawashty, S. A.; Saleh, N. A. M., Chemosystematic studies on certain species of the family Brassicaceae (Cruciferae) in Egypt. *Biochemical Systematics and Ecology* **2010,** 38, (4), 680-685.
29. Jiang, M.-Y.; Lu, H.; Pu, X.-Y.; Li, Y.-H.; Tian, K.; Xiong, Y.; Wang, W.; Huang, X.-Z., Laxative Metabolites from the Leaves of Moringa oleifera. *Journal of Agricultural and Food Chemistry* **2020,** 68, (30), 7850-7860.
30. Song, L.; Morrison, J. J.; Botting, N. P.; Thornalley, P. J., Analysis of glucosinolates, isothiocyanates, and amine degradation products in vegetable extracts and blood plasma by LC–MS/MS. *Analytical Biochemistry* **2005,** 347, (2), 234-243.
31. Yin, Z.; Zhang, W.; Feng, F.; Zhang, Y.; Kang, W., α-Glucosidase inhibitors isolated from medicinal plants. *Food Science and Human Wellness* **2014,** 3, (3-4), 136-174.
32. Lu, C.-H.; Liu, S.-S.; Wang, J.-Y.; Wang, M.-Z.; Shen, Y.-M., Characterization of Eight New Secondary Metabolites from the Mutant Strain G-444 of Tubercularia sp. TF5. *Helvetica Chimica Acta* **2014,** 97, (3), 334-344.
